# Genome-Wide Identification of bHLH Transcription Factors and Screening of Candidate Regulators Associated with Flavonoid Biosynthesis in *Platycodon grandiflorus* Under Methyl Jasmonate Treatment

**DOI:** 10.3390/genes17091144

**Published:** 2026-09-18

**Authors:** Yalan Feng, Runlong Wei, Yeying Wu, Dandan Cheng, Zhao Zhao, Chao Ma

**Affiliations:** 1 College of Life Sciences, Wuchang College of Technology, Wuhan 430200, China; 2 College of Agriculture, Henan University of Science and Technology, Luoyang 471023, China

**Keywords:** *P. grandiflorus*, bHLH transcription factor, flavonoid biosynthesis, genome-wide identification, expression analysis, regulatory factor

## Abstract

**Background**: The bHLH transcription factor family plays crucial roles in plant secondary metabolism, yet its genome-wide characterization and regulatory functions in flavonoid biosynthesis remain unexplored in *Platycodon grandiflorus*, a medicinal species with bioactive flavonoids. This study aims to systematically identify *PgbHLH* genes and screen for candidate regulators of flavonoid production. **Methods**: We performed genome-wide identification of bHLH family members using curated reference genomes, followed by phylogenetic classification, chromosomal localization, gene structure analysis, and collinearity assessment. Promoter cis-element prediction and subcellular localization assays for selected proteins were conducted. Expression profiles under methyl jasmonate (MeJA) treatment were evaluated via transcriptome sequencing and RT-qPCR. Protein–protein interaction prediction, expression correlation, and weighted gene co-expression network analysis (WGCNA) were integrated to link *PgbHLH* genes with flavonoid structural genes. **Results**: A total of 95 *PgbHLH* genes were identified and clustered into 23 subfamilies, unevenly distributed across nine chromosomes. All members retained conserved bHLH domains but varied in exon–intron structures. Thirty-six duplication events were detected, with Ka/Ks ratios indicating purifying selection. Promoters harbored multiple hormone- and stress-responsive elements. PgbHLH37, PgbHLH42, and PgbHLH70 were confirmed to be nuclear-localized. Transcriptomic and RT-qPCR data showed differential expression of *PgbHLH* genes in response to MeJA. Integrative analyses identified 23 PgbHLH members associated with flavonoid biosynthetic genes, among which PgbHLH37, PgbHLH42, and PgbHLH70 exhibited strong multi-dimensional correlations and were nominated as high-priority candidate regulators. **Conclusions**: This systematic characterization provides a foundation for understanding bHLH-mediated flavonoid metabolism in *P. grandiflorus*, with three prioritized candidates for future functional validation. The findings are strictly supported by the presented genomic and expression evidence. However, our conclusions are based on total flavonoid content, and analysis of individual flavonoid species is required to determine pathway-specific regulation.

## 1. Introduction

The basic helix-loop-helix (bHLH) gene family is the second largest family of transcription factors (TFs) in eukaryotes [1]. Members of the bHLH TF family contain 50–60 highly conserved amino acid residues, which can be structurally divided into two regions. The N-terminal region consists of approximately 10–15 amino acids and contains a conserved DNA-binding domain that recognizes the E-box motif (CANNTG). The C-terminal region comprises the HLH domain, which facilitates the formation of homodimers or heterodimers with other TFs, thereby enabling the regulation of target gene expression [2,3].

The bHLH family was first identified in animals and subsequently classified into six distinct groups (Groups A–F) based on their dimerization properties, tissue distribution patterns, and DNA-binding characteristics [4]. Plant bHLH TFs were initially discovered in maize [5,6], followed by the identification of *bHLH* genes in *Arabidopsis* [7], rice [8], wheat [9], and other crop species. In most plant species, bHLH proteins are predominantly associated with Group B [10]. Within plants, bHLH proteins commonly function as dimers within complex molecular regulatory networks, playing essential roles in various biological processes, including growth and development, secondary metabolite biosynthesis, and responses to biotic and abiotic stresses [11]. Secondary metabolite biosynthesis encompasses the production of flavonoids, terpenoids, alkaloids, and phenolic acids [12]. The biosynthesis pathway of flavonoids has been extensively characterized, and its various stages are subject to complex regulation, which determines the temporal, spatial, and biochemical specificity of their accumulation. MYC-like bHLH (avian Myelocytomatosis virus-like bHLH), R2R3-MYB (Repeat 2–Repeat 3 avian Myeloblastosis virus) TF and WDR (WD repeat or WD40 repeat) proteins play key roles. They form the MBW protein complex and directly regulate the expression of structural genes [13,14]. Anthocyanins, a subclass of flavonoids, serve as primary pigments in fruits and vegetables and also exhibit bioactive properties beneficial to human health. The TF bHLH interacts with MYB and WD40 proteins to form the MBW ternary complex, which regulates anthocyanin biosynthesis [15,16]. Research has demonstrated that PsbHLH1 positively regulates anthocyanin biosynthesis in peony and directly binds to the promoters of dihydroflavonol 4-reductase (PsDFR) and anthocyanin synthase (PsANS), thereby transcriptionally activating their expression [17]. Similarly, in mulberry, MabHLH3 acts as a key regulator of fruit coloration, and its aberrant expression disrupts the anthocyanin regulatory network, leading to alterations in pigment composition and fruit coloration [18]. In addition to activating numerous characterized downstream genes, bHLH proteins also exhibit repressive functions. For instance, the *CpbHLH1* gene in Cerasus inhibits anthocyanin accumulation in *Arabidopsis* [19]. The bHLH TF family influences plant responses to various stresses. Overexpression of the *bHLH035* gene in Populus euphratica enhanced drought tolerance through the regulation of stomatal movement and its subsequent impact on photosynthesis [20]. In pepper, the *CabHLH035* gene improved salt stress tolerance by modulating intracellular Na^+^/K^+^ homeostasis and promoting proline biosynthesis [21]. Transgenic tomato plants overexpressing the *IbbHLH79* gene demonstrate enhanced cold tolerance [22]. Methyl jasmonate (MeJA), a derivative of jasmonic acid, serves as an important regulatory molecule involved in plant growth and the biosynthesis of secondary metabolites. Previous studies have demonstrated that Methyl Jasmonate (MeJA) can induce the expression of bHLH in *P. grandiflorus*, leading to increased saponin accumulation and the regulation of downstream structural genes [23,24].

*P. grandiflorus*, a perennial medicinal and edible herb native to East Asia, possesses roots that are rich in diverse bioactive compounds—particularly triterpenoid saponins and flavonoids—and exhibit well-documented pharmacological activities, including antitussive, antiasthmatic, antitumor, antioxidant, and hepatoprotective effects [25]. Flavonoids constitute a critical class of secondary metabolites underpinning the plant’s therapeutic efficacy [26]. The bHLH TF family—the second largest TF family in plants after MYB—is extensively implicated in regulating plant growth and development, secondary metabolism, and abiotic/biotic stress responses [1,27]. Nevertheless, a comprehensive genome-wide identification and functional characterization of bHLH genes in *P. grandiflorus* remains lacking. Therefore, in this study, we performed systematic, whole-genome identification of bHLH TFs in *P. grandiflorus* and conducted integrative bioinformatic analyses encompassing physicochemical properties, phylogenetic evolution, exon–intron architecture, chromosomal localization, synteny relationships, and cis-regulatory elements within promoter regions. Furthermore, leveraging transcriptome profiling and RT-qPCR validation, the dynamic expression patterns of bHLH genes under multiple phytohormone and abiotic stress treatments were characterized. By integrating weighted gene co-expression network analysis (WGCNA), predicted protein–protein interaction networks, and expression correlation with key flavonoid biosynthetic genes, the candidate hub bHLH regulators potentially governing flavonoid accumulation were identified. Collectively, this work establishes a foundational genomic and functional framework for elucidating the mechanistic roles of bHLH TFs in the regulation of flavonoid biosynthesis in *P. grandiflorus*.

## 2. Materials and Methods

### 2.1. Plant Materials and Treatment

The experimental material, *P*. *grandiflorus* (Jacq,) A. DC. cv. Luyuan Jiegeng 1, is a registered medicinal cultivar of *P. grandiflorus* in Shandong Province, China. Seeds of the cultivar were obtained from the breeding unit of Shandong Academy of Agricultural Sciences/the germplasm repository of the Shandong Provincial Chinese Herbal Medicine Industrial System in 2024 and were multiplied for two generations under uniform field conditions at our experimental station under standard field conditions before sampling. Seedlings were cultivated at 25 °C under a photoperiod of 14 h/10 h and a light intensity of 120 μmol·m^−2^·s^−1^. After 90 days of growth, the plants were treated with 100 μmol·L^−1^ abscisic acid (ABA), indole-3-acetic acid (IAA), gibberellic acid (GA), and methyl jasmonate (MeJA), as well as with 20% PEG-6000 (to simulate drought), low temperature (4 °C), high temperature (38 °C), and 200 mmol·L^−1^ NaCl (salt stress), while normally growing plants were used as controls. Three independent biological replicates were performed for each treatment and the control group, with each replicate consisting of roots pooled from three individual plants. Whole roots from each treatment group were collected at 0, 12, 24, and 48 h after treatment and immediately frozen in liquid nitrogen for storage. Pooling of roots from three individuals per replicate was performed to minimize individual variation; however, we recognize that this approach does not allow assessment of inter-individual variability.

### 2.2. Bioinformatics Analysis

#### 2.2.1. Identification of PgbHLH TF Family Members

The genomic and annotation data of *P. grandiflorus*, including the genome sequence, protein sequences, and gene annotation file (GFF), were retrieved from the Genome Warehouse (GWH) database (https://ngdc.cncb.ac.cn/gwh/) (accessed on 21 April 2025) under accession number GWHARYT00000000. To exhaustively and impartially identify all members of the bHLH family, we adopted a dual-strategy approach combining HMM-based and homology-based searches. For the HMM search, the HLH domain profile (PF00010) from Pfam was used with HMMER version 3.3.2, applying an E-value threshold of 0.01 against the *P. grandiflorus* proteome. In parallel, a BLASTP search was performed using all bHLH protein sequences from *Arabidopsis thaliana* (obtained from TAIR, https://www.arabidopsis.org/) (accessed on 1 March 2025) as queries, with the same E-value cutoff. Candidate sequences from both approaches were pooled, and redundant entries were eliminated. For validation, the protein sequences of the initial candidates were submitted to the NCBI CDD tool and the Pfam database; sequences lacking a discernible HLH domain were discarded. After sequence alignment, those with obvious defects (e.g., incomplete or erroneous regions) were manually removed, and only genes harboring a complete bHLH domain were retained. This rigorous workflow guarantees the high-confidence identification of the PgbHLH family.

#### 2.2.2. Phylogenetic Tree Construction of bHLH TF Family Members in *P. grandiflorus*

To establish a phylogenetic framework for the bHLH family, we performed multiple sequence alignment of bHLH proteins from *P. grandiflorus* and *A. thaliana* using MEGA11 software (Version 11.0.13; Mega Software, Philadelphia, PA, USA). The phylogenetic tree was constructed using the Neighbor-Joining (NJ) method with the p-distance model, and bootstrap values were obtained from 1000 replicates.

#### 2.2.3. Analysis of Physical Properties and Domain Characteristics of the bHLH TF Family Members in *P. grandiflorus*

The bHLH proteins in *P. grandiflorus* were analyzed using the ExPASy tool (www.ExPASy.org/) (accessed on 21 April 2025), including molecular weight, amino acid quantity, and instability index, and the subcellular localization was predicted by WoLF PSORT (https://wolfpsort.hgc.jp/) (accessed on 21 April 2025). The secondary and tertiary structures of the bHLH proteins were forecasted via the websites NPS-SOPMA (https://npsa.lyon.inserm.fr/cgi-bin/npsa_automat.pl?page=/NPSA/npsa_sopma.html) (accessed on 21 April 2025) and SWISS-MODEL (https://swissmodel.expasy.org/) (accessed on 21 April 2025). Multi sequence alignment of bHLH protein in *P. grandiflorus* was executed through Jalview software (Version 2.11.2.4), and the alignment results were visualized by means of the SeqLogo tool in TBtools software (Version 2.481; South China Agricultural University, Guangzhou, China).

#### 2.2.4. Gene Structure and Conserved Motif Analysis of bHLH TF Family in *P. grandiflorus*

The gene structure was analyzed using the gene annotation file and visualized via TBtools. The exon–intron structure of these TFs was determined through GSDS (version 2.0) [28]. The conserved motifs of the PgbHLH protein were identified according to MEME (version 4.9.1). Finally, the motif patterns were presented using the TBtools software [29].

#### 2.2.5. Chromosome Localization, Ka/Ks, and Collinearity Analysis of the bHLH TF Family in *P. grandiflorus*

The chromosome localization of the *P. grandiflorus* bHLH TF family was performed via MapChart v.2.3 software (version 2.3). The intra- and interspecific homology and collinearity of the PgbHLH TF family members were analyzed using MCScanX [29], and visual analysis was conducted through the software [30]. Then, gene pairs with collinearity were selected, and the Ka/Ks values were calculated by TBtools for stress selection analysis [31].

#### 2.2.6. Analysis of Cis-Regulatory Elements in the PgbHLH TF Family

The cis-regulatory elements in the upstream sequence (2 kb) of the start codon of the *PgbHLH* gene were predicted by the PlantCARE online tool, and the cis-elements related to abiotic stress were visualized through TBtools.

### 2.3. Experimental Analysis

#### 2.3.1. Determination of Total Flavonoids and Transcriptome Sequencing in *P. grandiflorus* Treated with Exogenous MeJA

The content of total flavonoids in whole roots of 100 μmol/L MeJA-treated samples was determined according to the method of Y. Y. Cao et al. [23]. Another portion was extracted with Trizol (Takara, 9108Q, manufactured by Baosheng Bioengineering (Dalian) Co., Ltd., Dalian, China) reagent, and the transcriptome was sequenced by Beijing Biomarker Technologies Co., LTD. (Beijing, China). with normal growth as the control. Transcriptome sequencing was conducted exclusively for MeJA-treated samples, whereas the other hormone and stress treatments were applied only for RT-qPCR validation of candidate genes. The FPKM values were calculated and converted via Log_2_(FPKM + 1), and an expression heat map of *PgbHLH* genes’ FPKM values was plotted using TBtools software. Flavonoid content data are presented as mean ± SD of three biological replicates. Comparisons between MeJA-treated samples and the 0 h control at each time point were assessed by Student’s *t*-test (two-tailed), with *p* < 0.05 considered statistically significant.

#### 2.3.2. Prediction of Interaction Between PgbHLH Protein and Flavonoid Biosynthesis Structural Gene Protein and Analysis of Expression Level Correlation

Based on the sequencing data of *P. grandiflorus* under MeJA treatment, the expression patterns of flavonoid synthesis-related structural genes were screened out, and then their correlation with the expression level of the *bHLH* gene was analyzed. Finally, the results with significant correlations were presented in the form of a heat map using TBtools. Interactions were predicted using STRING (https://www.string-db.org/) (accessed on 21 April 2025) based on homologous *Arabidopsis* proteins; these predictions are computational and require experimental validation.

#### 2.3.3. Screening and WGCAN Analysis of Differentially Expressed Genes in Transcriptome

The expression levels of all genes were standardized by FPKM, and further converted to TPM values for subsequent comparison. The differentially expressed genes (DEGs) were screened with |log_2_FC| ≥ 1 and FDR < 0.05 as the threshold, and an enrichment analysis of Gene Ontology (GO) annotation, Cluster of Orthologous Groups (COG) and the KEGG pathway was conducted. Through the WGCNA (v1.74) in R (v4.4.0), a weighted gene co-expression network system was constructed to analyze the co-expression relationship between genes. Taking the expression matrix converted from log_2_(TPM + 1) as the input, by selecting a soft-threshold power of β = 15 (at this time, the scale-free network fitting index was R^2^ = 0.8), the topological overlap matrix (TOM) conforming to the scale-free distribution was constructed. On this basis, the dynamic tree pruning algorithm was used to identify the co-expression module (the minimum number of module genes was set to 100), and the module characteristic genes were calculated. Finally, the core genes of each module were screened according to the correlation between genes and module characteristic genes to reveal the potential key regulatory factors.

#### 2.3.4. Subcellular Localization of Candidate Genes in *N. benthamiana* Leaf Epidermal Cells

To examine the subcellular localization of PgbHLH37, PgbHLH42, and PgbHLH70, which are putatively involved in the regulation of flavonoid biosynthesis in *P. grandiflorus*, we adopted the protocol of C. Sun et al. [32]. The full-length coding sequences were PCR-amplified and inserted into the pCAMBIA1300-GFP vector (without the stop codon), yielding the fusion constructs PgbHLH37-GFP, PgbHLH42-GFP, and PgbHLH70-GFP. These recombinant plasmids were then introduced into *Agrobacterium* strain GV3101. The transformed cells were cultured to an OD_600_ of approximately 0.8, pelleted by centrifugation, resuspended to the same density, and infiltrated into the epidermal cells of *N. benthamiana* leaves. After infiltration, nuclei were counterstained with DAPI (4′,6-diamidino-2-phenylindole). Nuclear localization was assessed by the overlap of GFP fluorescence with DAPI signals, and the green fluorescence of the fusion proteins was visualized under a confocal laser scanning microscope. The cDNA template was synthesized from total RNA extracted from *P. grandiflorus* whole roots, and the specific gene sequences were retrieved from the genomic data described in Section 2.2.1.

#### 2.3.5. Extraction of Total RNA from *P. grandiflorus*, Synthesis of cDNA and RT-qPCR Detection

Whole roots were subjected to treatments with 100 μmol/L of ABA, IAA, GA, MeJA, and 20% PEG-6000 (simulating drought) at 4 °C (low temperature) and 38 °C (high temperature), and 200 mmol/L NaCl (salt), for a duration of 12 h. In this study, 100 μmol/L was selected as the treatment concentration, as this concentration has been demonstrated to be effective in inducing bHLH transcription factor responses and secondary metabolite accumulation in various medicinal plants [33,34]. The treatment duration of 12 h was chosen based on the early and rapid activation characteristics of transcription factors in response to exogenous signals; bHLH and other transcription factors often exhibit significant expression changes within hours of hormonal or stress treatments [35,36]. Hence, the 12 h time point effectively captures the early transcriptional responses of bHLH transcription factors to diverse exogenous stimuli, while also ensuring the feasibility of parallel comparisons across different treatments.

Subsequently, RNA was extracted using Trizol (Takara, 9108Q) reagent and reverse-transcribed into cDNA (TaKaRa, RR047Q) for subsequent RT-qPCR analysis. The RT-qPCR reaction system and procedure were established using the instruction manual of the ChamQ Universal SYBR qPCR master Mix (Vazyme, Nanjing No.1 Zonjian Biotechnology Co., Ltd., Nanjing, China), and the relative expression levels were calculated using the 2^−ΔΔCt^ method [31]. Four *PgbHLH* genes with significantly down-regulated expression, one with complete non-expression, and four with significantly up-regulated expression were selected based on the RNA-seq data. The selected genes were chosen to cover the full spectrum of expression changes observed in the RNA-seq data (|log_2_FC| ≥ 1 and FDR < 0.05), including the most significantly up-regulated and down-regulated members, as well as one gene with no detectable expression, to ensure a balanced validation set. Relative expression levels were calculated using the 2^−ΔΔCt^ method. Statistical significance between treated and control samples was assessed by Student’s *t*-test (two-tailed, unequal variance) using three biological replicates each with three technical replicates; *p* < 0.05 was considered statistically significant. Primers were designed via Primer Premier 5 (Table 1), with *PgActin* as the internal reference gene.

## 3. Results

### 3.1. Identification and Phylogenetic Analysis of the bHLH Gene Family in P. grandiflorus

**Figure 1:**
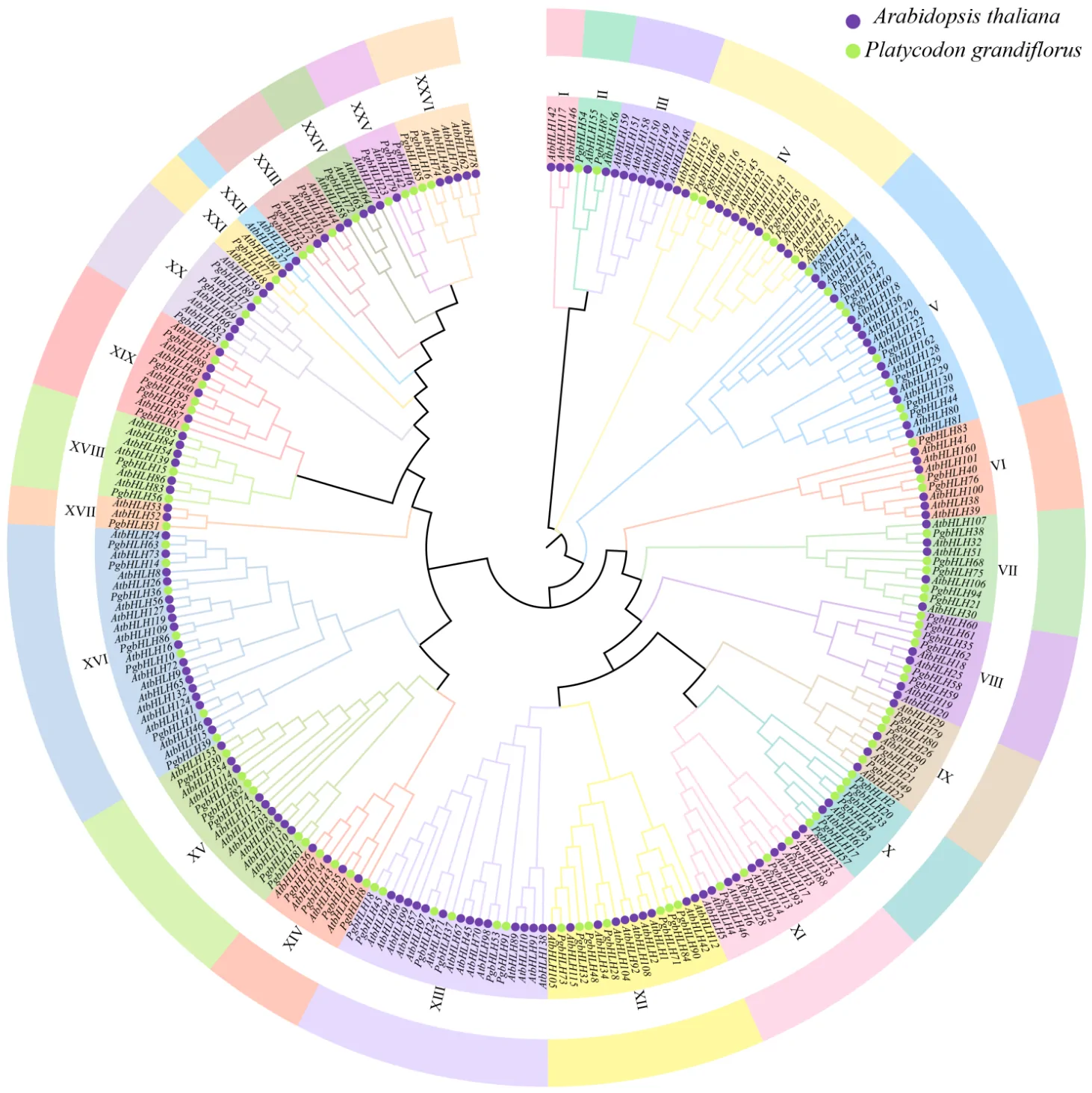
Phylogenetic tree of *bHLH* gene family members in *Platycodon grandiflorus* and *Arabidopsis thaliana.* Phylogenetic tree of bHLH proteins constructed using the Neighbor-Joining (NJ) method with the p-distance model based on multiple sequence alignments of bHLH proteins from *P. grandiflorus* and *A. thaliana* (MEGA11; 1000 bootstrap replicates). The tree includes 161 AtbHLH and 95 PgbHLH sequences. According to the classification of the *A. thaliana* bHLH family, all sequences are grouped into 26 subfamilies; 95 PgbHLH proteins are distributed across 23 subfamilies, with no members in subfamilies I, III, and XXII, whereas AtbHLH members are present in all 26 subfamilies. In most subfamilies, the number of *AtbHLH* genes exceeds that of *PgbHLH* genes, except for subfamilies II, VII, IX, XIV, XIX, XXIII, and XXIV, where the gene numbers are similar between the two species.

To investigate the evolutionary relationships and classification of the *PgbHLH* members, 161 AtbHLH protein sequences from *A. thaliana* were taken as target sequences. The candidate genes were compared with *A. thaliana* through NCBI, and genes with complete conserved bHLH domains were selected. Eventually, 95 members of the PgbHLH TFs were obtained. The genes were renamed according to their chromosomal locations, namely *PgbHLH1* to *PgbHLH95*. A phylogenetic tree was constructed using 161 AtbHLH protein sequences and 95 PgbHLH protein sequences, as shown in Figure 1. According to the classification of bHLH proteins in *A. thaliana*, all sequences were divided into 26 subfamilies. Among them, 95 PgbHLH proteins clustered in 23 subfamilies, and there were no PgbHLH family members in the I, III, and XXII subfamilies. The absence of PgbHLH members in subfamilies I, III, and XXII may reflect genuine evolutionary loss or insufficient sequence divergence for clustering; further investigation is needed. All subfamilies had AtbHLH family members in *A. thaliana*. Among the 26 subfamilies, the number of *AtbHLH* genes in most subfamilies was greater than that of *PgbHLH* genes. The *PgbHLH* genes in subfamilies II, VII, IX, XIV, XIX, XXIII, and XXIV were similar in number to the *AtbHLH* genes. The classification follows the *Arabidopsis* scheme as a reference framework, as no dedicated *P. grandiflorus*-specific classification system is currently available. We acknowledge that lineage-specific clades may exist but were not resolved due to the lack of a dedicated *P. grandiflorus*-specific classification. Bootstrap values ≥70% were considered reliable; nodes with lower support should be interpreted with caution.

### 3.2. Physicochemical Properties and Subcellular Localization Prediction of bHLH Family Proteins in P. grandiflorus

The physicochemical properties of the *PgbHLH* gene family members were analyzed using TBtools (Supplementary Table S1). The results showed that the amino acid range of the 95 *PgbHLH* family members was 71 to 781. The relative molecular mass was 8.02 kD to 86.35 kD. The isoelectric point range was 4.59 to 10.22. Among them, 67 (70.52%) proteins were acidic and 28 (29.48%) were basic. The protein instability index range was 35.28 to 86.33, and only 6 proteins had an instability index less than 40. The high instability indices of most proteins are common for transcription factors, which often undergo rapid turnover.

The fat coefficient range was 55.17 to 105.47, indicating good thermal stability of the *PgbHLH* proteins. The average hydrophobic indices of the PgbHLH protein were all less than 0, indicating that it is a hydrophilic protein. The subcellular localization prediction results revealed that 84 (88.42%) PgbHLH proteins were predicted to be located in the nucleus; PgbHLH26, PgbHLH41, PgbHLH48 and PgbHLH67 were predicted to be located in the cytoplasm; PgbHLH29, PgbHLH39, PgbHLH57 and PgbHLH69 were predicted to be located in the chloroplast; and PgbHLH30, PgbHLH43 and PgbHLH70 were predicted to be located in the peroxisome, mitochondrion and chloroplast respectively, indicating that most of the PgbHLH proteins are predicted to be localized in the nucleus, which is consistent with their expected function as transcription factors. A few proteins were predicted to localize in chloroplasts, mitochondria, or peroxisomes; these predictions may indicate alternative functions or prediction errors, and require experimental verification.

### 3.3. Analysis of the Gene Structure and Conserved Motifs of Members of the PgbHLH TF Family

**Figure 2:**
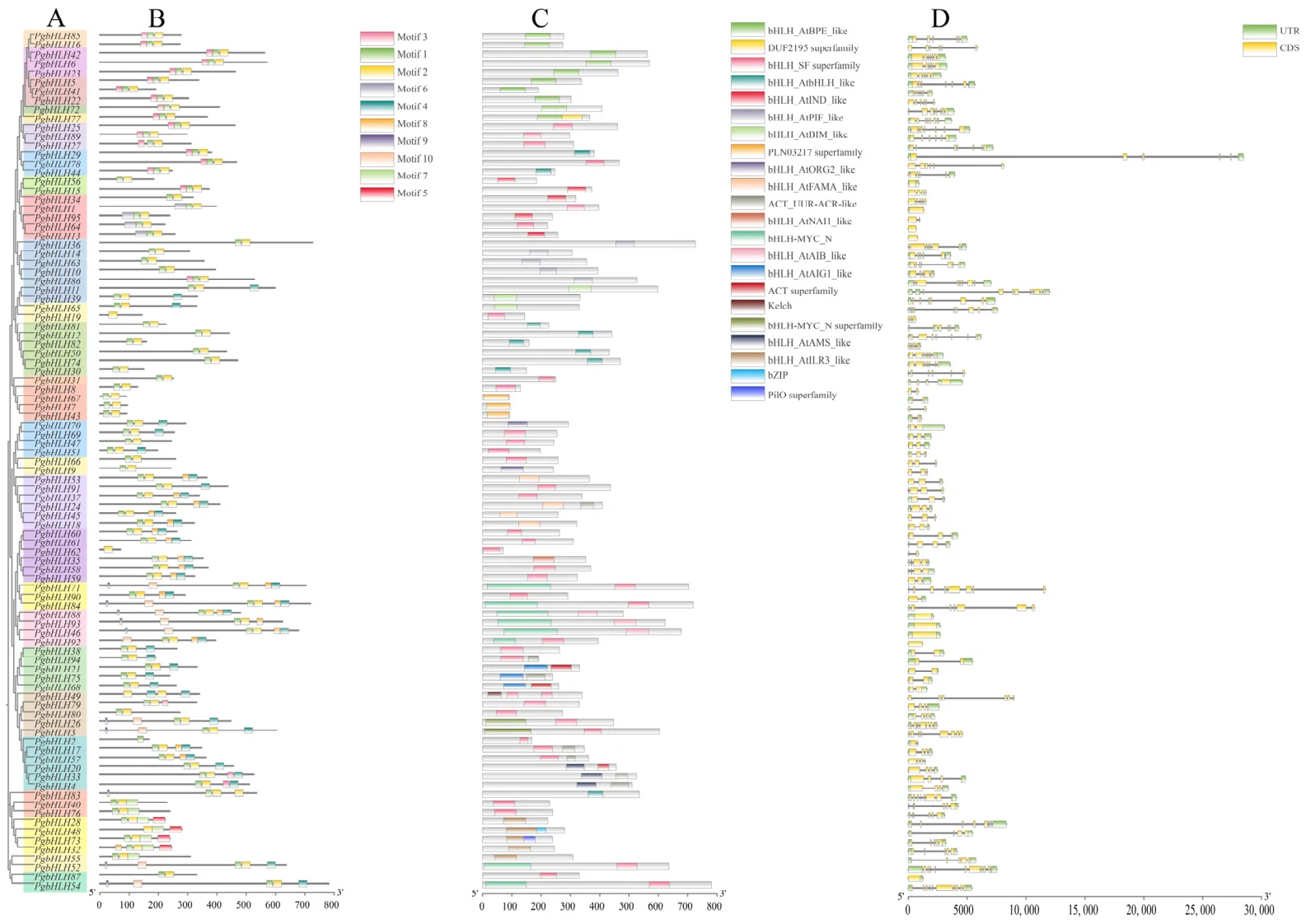
Phylogenetic relationship, conserved domain, protein motif and gene structure of *bHLH* gene in *P. grandiflorus.* (**A**) Phylogenetic tree of PgbHLH proteins. (**B**) Distribution of conserved motifs in PgbHLH proteins. (**C**) Conserved bHLH domain organization of PgbHLH proteins. (**D**) Exon–intron structure of *PgbHLH* genes.

The evolution of multi-gene families generally results from genetic structural diversity. To describe the genetic structural diversity of the *PgbHLH* gene, the exon–intron structure was analyzed (Figure 2). As shown in Figure 2D, the number of introns in the *PgbHLH* gene ranges from 0 to 13, and three *PgbHLH* genes (*PgbHLH64*, *PgbHLH13*, and *PgbHLH95*) had no introns. All members of the *PgbHLH* gene family contained exons, and 13 *PgbHLH* genes had no untranslated regions (UTR). These differences may be part of the reason for the functional differentiation of this gene family. The domain structure of the *PgbHLH* gene is exhibited in Figure 2C. All *PgbHLH* genes contained the bHLH-related domain and genes with the same bHLH domain were located in the same or adjacent subfamilies. The MEME suite (https://meme-suite.org/meme/tools/meme) (accessed on 21 April 2025) identified 10 conserved motifs in the PgbHLH protein (Figure 2B). All members of the *PgbHLH* gene contain Motif 2, Motif 3 exists only in the XX~XXVI subfamily of the *PgbHLH* gene, and Motif 5 exists only in the XII subfamily of the *PgbHLH* gene. This indicated that the protein variations were diverse, and the same or adjacent subfamilies had the same conserved motifs. The presence of intronless genes and missing UTRs may reflect retrotransposition events or functional diversification, but further investigation is required to confirm these possibilities. The motif conservation patterns are described qualitatively, and we acknowledge that statistical enrichment tests were not performed.

### 3.4. Chromosome Localization and Collinearity Analysis of the bHLH Gene Family in P. grandiflorus

**Figure 3:**
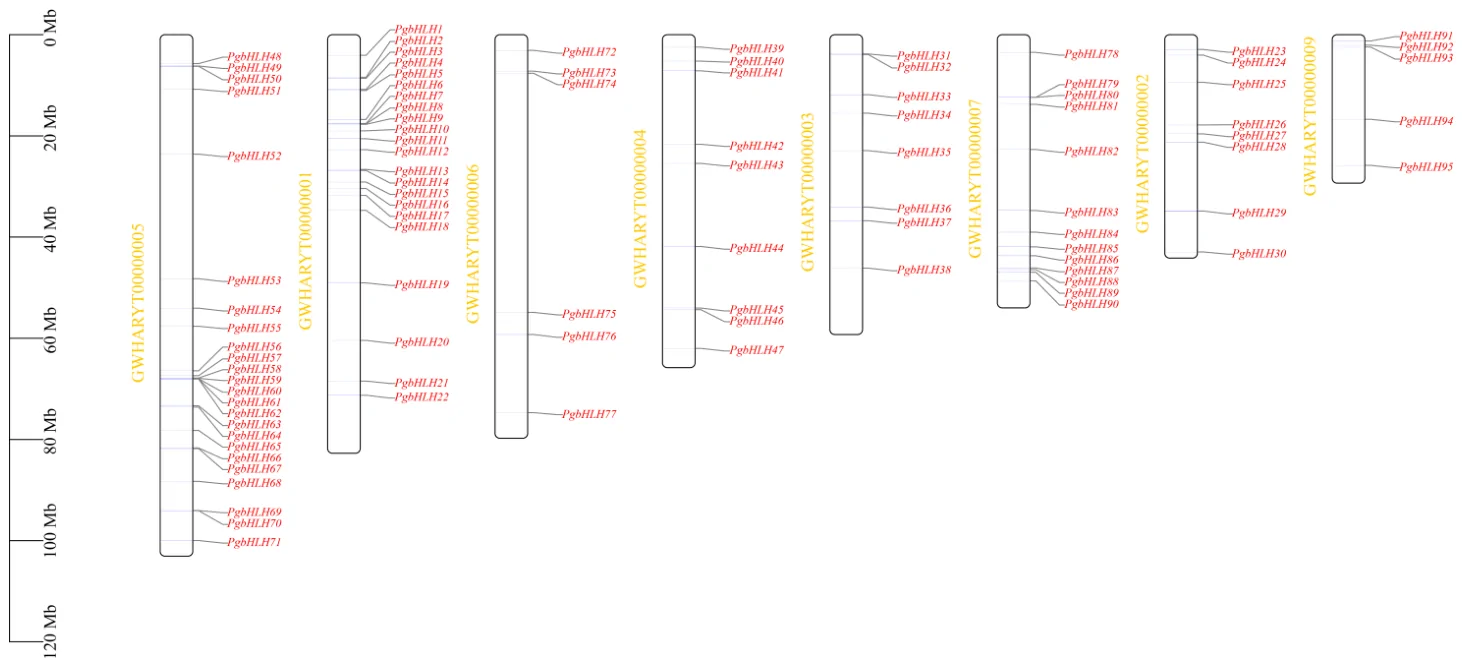
Chromosomal localization of *bHLH* gene in *P. grandiflorus.*

A chromosome localization map of the *PgbHLH* genes in *P. grandiflorus* was drawn through the TBtools software (Figure 3). The results indicated that the 95 gene members were located on nine chromosomes. The number of *PgbHLH* genes on each chromosome ranged from 5 to 24. Chromosome 5 had the largest number of *PgbHLH* genes, with 24, while chromosome 9 had the smallest number, with 5. Additionally, except for chromosome 2, the members were clustered on the other chromosomes, suggesting the possibility of gene duplication. The uneven distribution of *PgbHLH* genes across chromosomes may reflect segmental duplication events that contributed to the expansion of this gene family on certain chromosomes.

**Figure 4:**
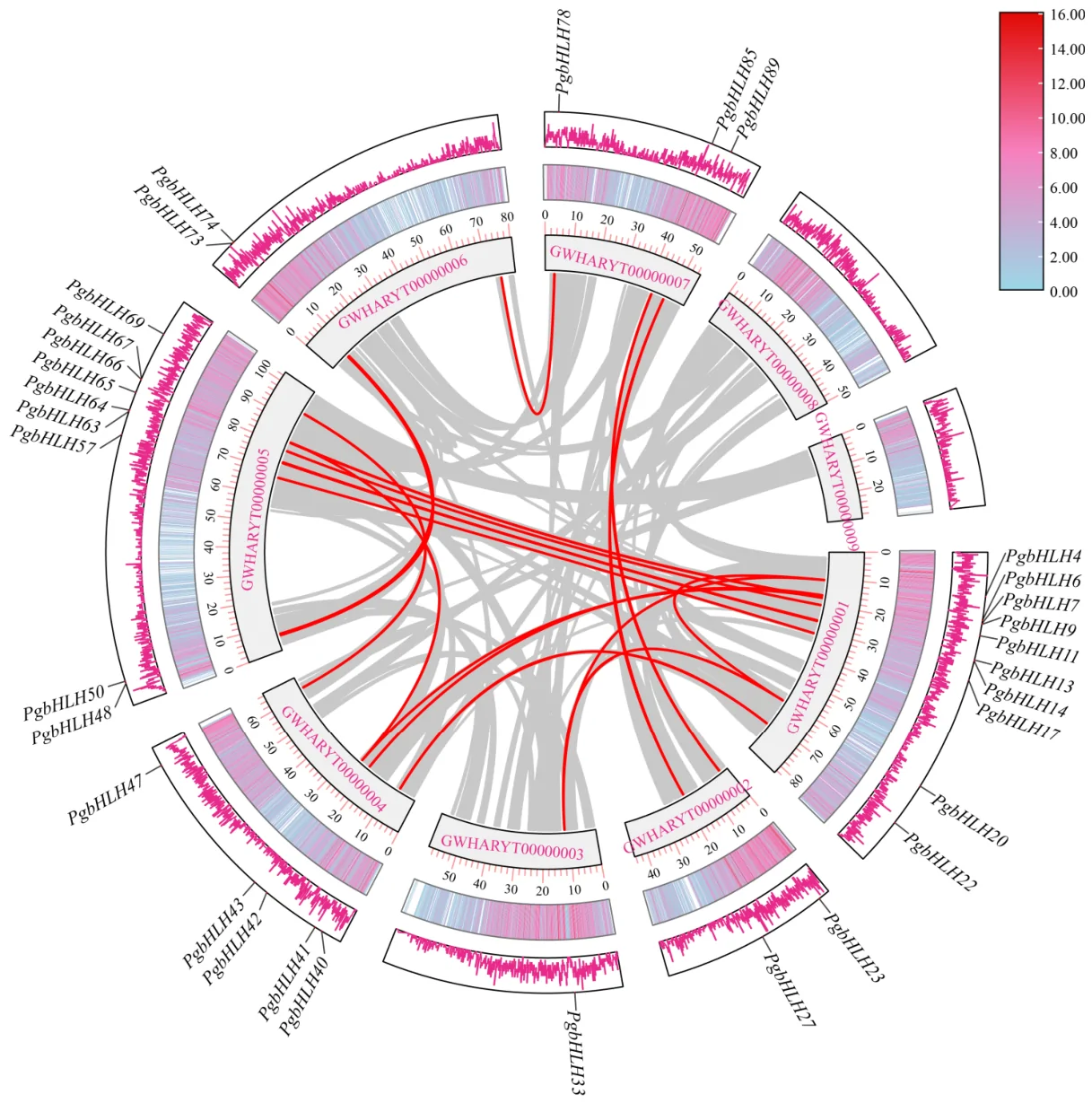
Collinearity analysis of the *bHLH* gene region in *P. grandiflorus.* Circles represent the nine chromosomes; gray lines indicate genomic syntenic blocks, and red lines highlight duplication events among *PgbHLH* genes. A total of 36 duplicated gene pairs were identified, including three tandemly duplicated pairs (*PgbHLH13/14*, *PgbHLH66/67*, *PgbHLH73/74*) and 33 segmentally duplicated pairs. No duplication events were observed on chromosomes 8 and 9. The Ka/Ks ratios for all duplicated pairs were less than 1, indicating that these genes have undergone purifying selection during evolution.

**Figure 5:**
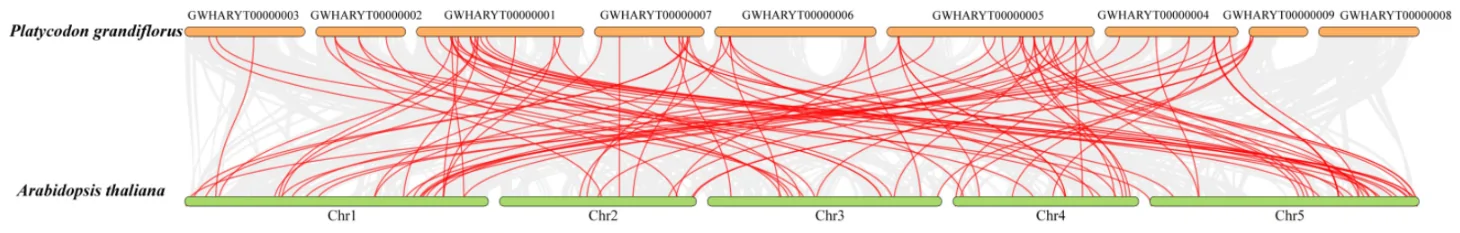
Analysis of interspecific collinearity between *Platycodon grandifloru* and *A. thaliana.* Gray lines indicate syntenic blocks between the two genomes, and red lines highlight orthologous *bHLH* gene pairs. A total of 102 orthologous pairs were identified, revealing a high degree of collinearity conservation for *bHLH* genes between the two species, suggesting structural stability of this gene family during evolution.

To determine the relationship between the *PgbHLH* gene and the repetitive events, the repetitive pattern of the *PgbHLH* gene was analyzed using BLASTP and MCscanX. As shown in Figure 4, seven chromosomes had 36 gene duplication events. No gene duplication events were observed on chromosomes 8 and 9. Among the 36 gene duplications, there were three pairs of tandemly duplicated genes (*PgbHLH13/14*, *PgbHLH66/67*, *PgbHLH73/74*), and 33 pairs of fragment duplications, indicating that fragment duplication play a major role in the evolution of the *PgbHLHs*. It can be seen from Figure 5 that a total of 102 *bHLH* genes of *Arabidopsis* and *P. grandiflorus* have linear homology, and the *bHLH* genes of Arabidopsis and *P. grandiflorus* are highly homologous. The high collinearity with Arabidopsis indicates conserved genomic architecture between the two species, but also suggests that some rearrangements may have occurred during evolution.

To understand the natural selection pressure that the repetitive genes of *PgbHLH* have undergone during evolution, the Ks values, Ka values, and Ka/Ks ratios were calculated (see Supplementary Table S2). The results showed that the Ka/Ks values of the 36 collinear gene pairs were all less than 1, indicating that they have all undergone purifying selection. This suggests that most *PgbHLHs* have evolved slowly.

### 3.5. Analysis of Cis-Acting Elements in the Promoter Region of the bHLH Gene of P. grandiflorus

**Figure 6:**
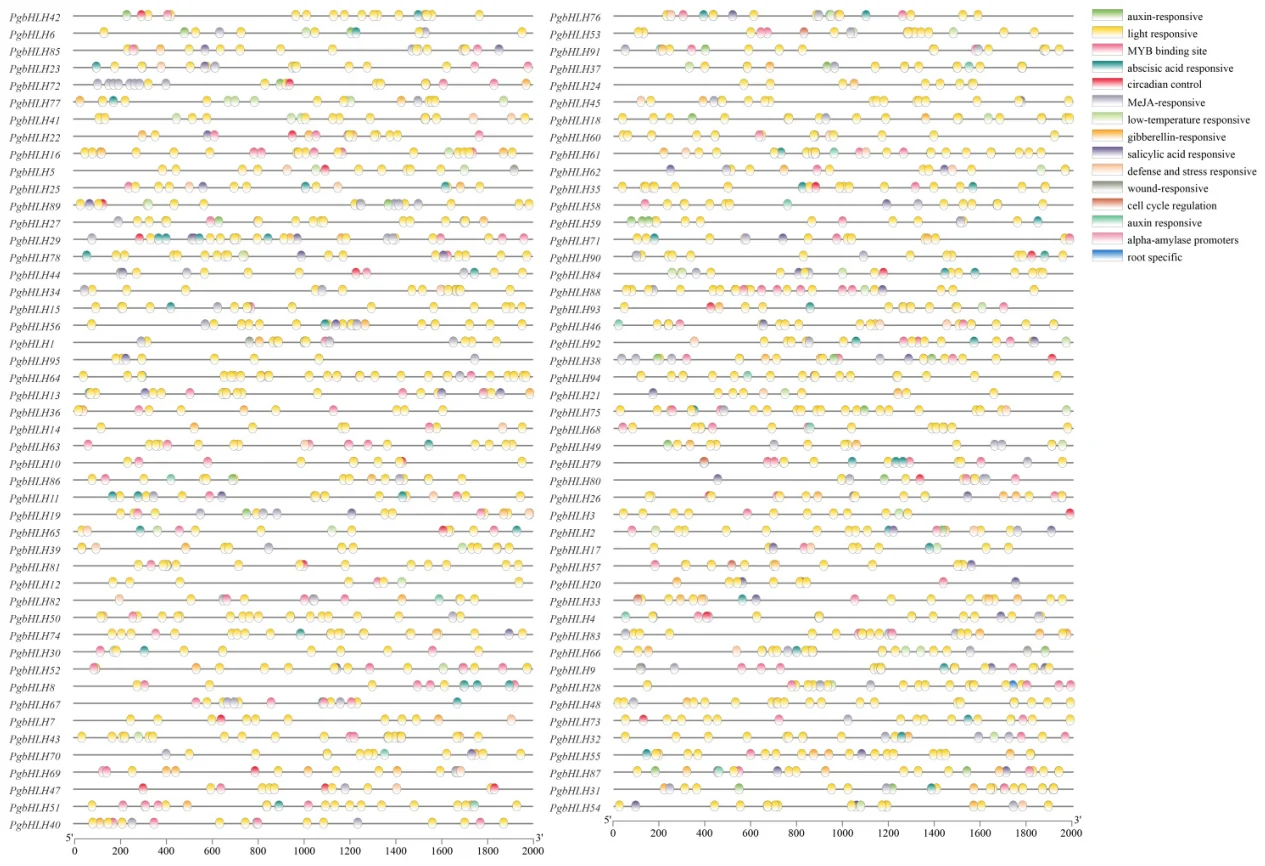
The cis-acting regulatory elements of the *bHLH* gene promoter in *P. grandiflorus.*

According to PlantCARE, the cis-elements within 2 kb upstream of the ATG initiation codon of 95 PgbHLH promoters were predicted. The results are presented in Figure 6. Hormone- and stress-related elements are overrepresented relative to random expectation, suggesting potential regulatory roles in phytohormone signaling and stress responses. As shown in Figure 6, all the genomic members of *PgbHLHs* contain elements related to light response. Many of the *PgbHLH* genomic members contain MYB binding sites, and some members contain response elements related to flavonoid biosynthesis. Among the hormone response elements, the members containing GA and ABA response elements were the most abundant, and the members containing IAA response elements were the fewest. Among the stress response elements, the members containing stress defense response elements were the most abundant. *PgbHLH28* was the only gene containing root-specific response elements. However, it should be noted that this cis-element analysis is purely computational and based on sequence prediction. Functional validation (e.g., promoter–reporter assays) is required to confirm the actual regulatory roles of these elements. We acknowledge that enrichment analysis was not performed.

### 3.6. Kinetic Pattern of Total Flavonoids in Response of P. grandiflorus to Exogenous MeJA Treatment

**Figure 7:**
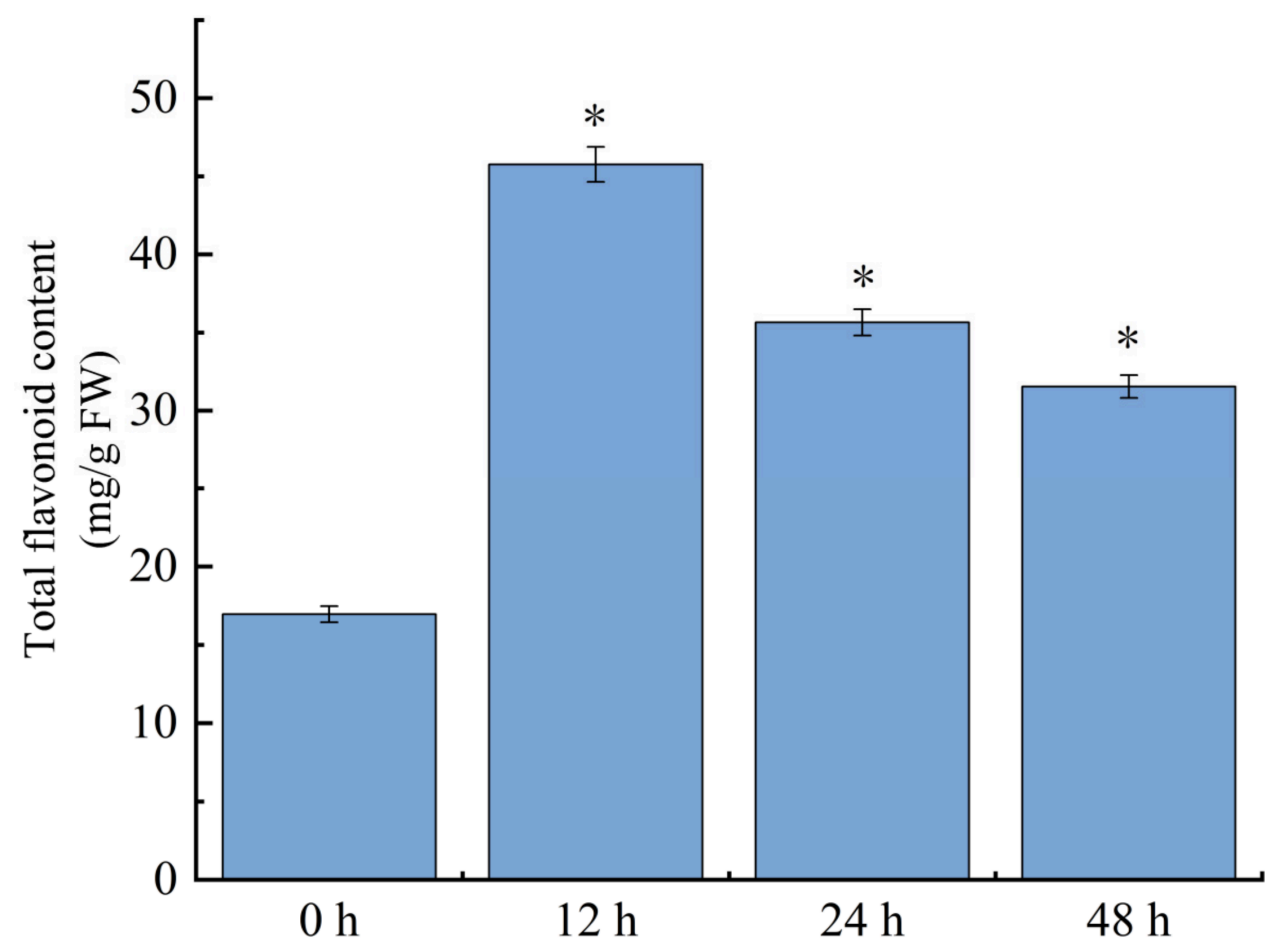
Dynamic changes in flavonoid content in whole roots of *P. grandiflorus* after MeJA treatment. Data are presented as mean ± SD (*n* = 3 biological replicates). Asterisks indicate significant differences compared to the 0 h control as determined by Student’s *t*-test (two-tailed): * *p* < 0.05.

To elucidate the kinetic profile of total flavonoid accumulation in whole roots of *P. grandiflorus* under exogenous MeJA treatment, this study quantified total flavonoid content at multiple time points post-treatment. As illustrated in Figure 7, flavonoid levels exhibited a biphasic response: an initial increase followed by a subsequent decline with prolonged exposure. The maximum accumulation (45.76 mg/g) was observed at 12 h post-treatment—representing a statistically significant 170.01% increase relative to the 0 h control. Although flavonoid content remained significantly elevated at 48 h compared with baseline, it declined by 32.61% relative to the 12 h peak. It should be noted that these measurements reflect total flavonoid content; quantification of individual flavonoid classes would be required to assess pathway-specific regulatory effects.

### 3.7. Transcriptome Profiling of P. grandiflorus Whole Roots in Response to Exogenous MeJA

**Figure 8:**
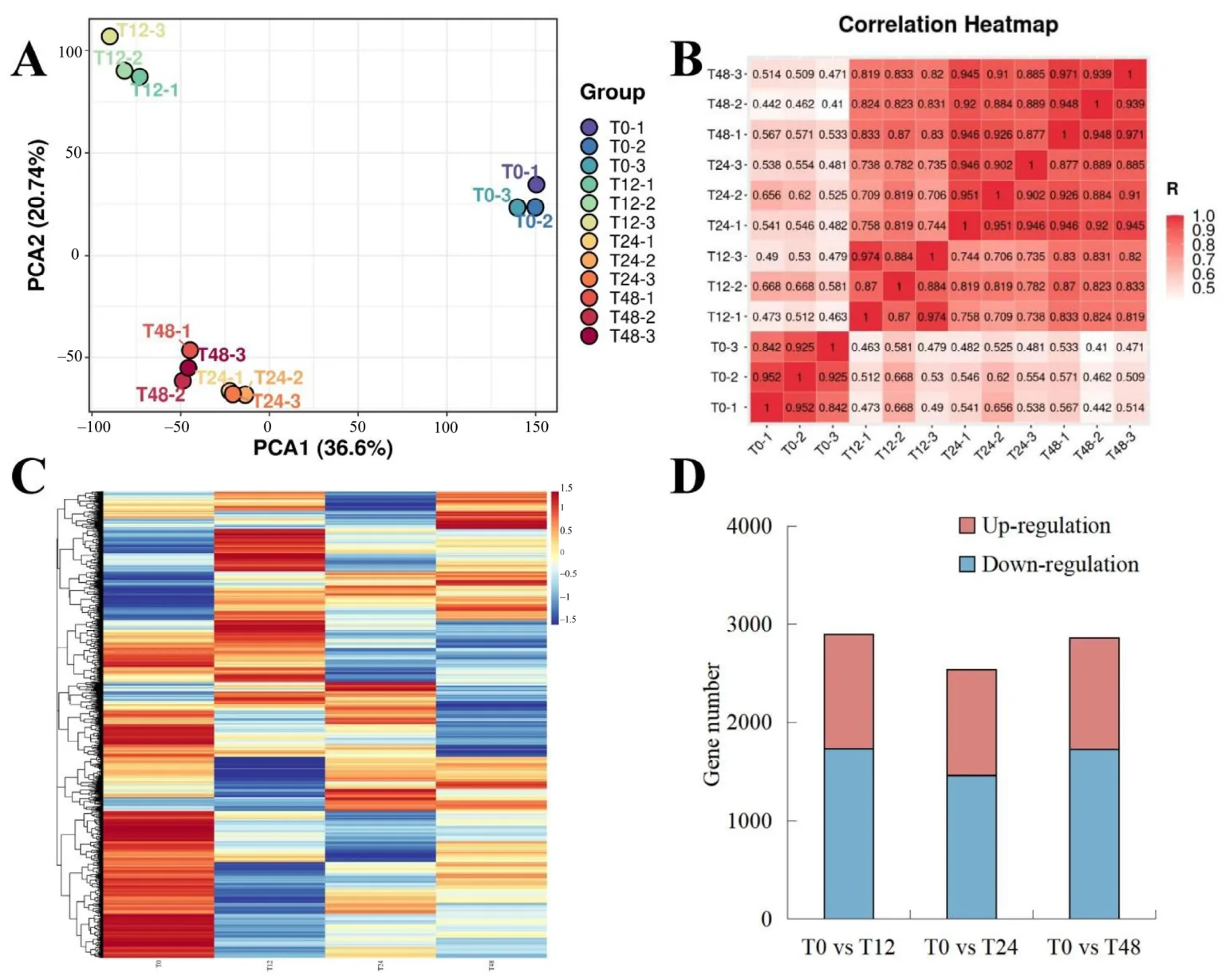
Transcriptome data analysis. (**A**) PCA. (**B**) Correlation heatmap. Each column represents a sample, and each row represents a gene. Different colors in the heatmap indicate the difference in expression levels. (**C**) Heatmap of gene expression levels in the root of *P. grandiflorus* under MeJA treatment. (**D**) The number of differentially expressed genes at 12 h, 24 h, 48 h and 0 h of MeJA treatment. T0, T12, T24 and T48 represent the samples of MeJA treatment at 0 h, 12 h, 24 h and 48 h respectively.

Principal component analysis (PCA) revealed distinct transcriptional divergence among *P. grandiflorus* root samples subjected to MeJA treatment across time points (Figure 8A). Clear separation was observed between the untreated control group and all MeJA-treated groups, indicating a robust transcriptional response to elicitation. Samples collected at 24 h and 48 h post-treatment exhibited greater similarity in PCA space—consistent with attenuated dynamic changes in flavonoid accumulation at later stages. The first two principal components (PC1 and PC2) collectively accounted for 57.34% of total variance (PC1: 36.60%; PC2: 20.74%). Hierarchical clustering analysis (HCA) of expression profiles (Figure 8B) further corroborated time-dependent modulation of flavonoid levels. Untreated controls displayed the lowest relative flavonoid abundance (lightest heatmap color), whereas peak accumulation occurred at 12 h post-MeJA treatment (darkest color). Flavonoid levels at 24 h and 48 h were intermediate and statistically indistinguishable from each other, supporting a transient induction profile. Collectively, PCA and HCA demonstrate that MeJA elicits a pronounced, temporally resolved reprogramming of flavonoid metabolism in *P. grandiflorus* whole roots. To dissect the underlying molecular mechanisms governing flavonoid biosynthesis, RNA-seq was performed on 12 cDNA libraries (one cultivar × four time points [T0, T12, T24, T48] × three biological replicates). A total of 25,855 annotated genes were quantified, and their expression dynamics were visualized in the clustered heat map (Figure 8C). High inter-replicate correlation confirmed sequencing data reliability and analytical suitability for downstream differential expression analysis. Pairwise comparisons against the untreated control (T0) identified 2984, 2578, and 2942 DEGs (|log_2_FC| ≥ 1 and FDR < 0.05) at T12, T24, and T48, respectively (Figure 8D). Notably, the magnitude and directionality of transcriptional responses were time-dependent: at T12 and T48, up-regulated DEGs totaled 1112 and 1131, respectively, while down-regulated DEGs totaled 1872 and 1811; in contrast, T24 exhibited the smallest transcriptional shift, with only 1004 up-regulated and 1574 down-regulated genes. This pattern aligns with the biphasic flavonoid accumulation kinetics observed at the transcriptional level, suggesting coordinated transcriptional regulation underlying the early induction and subsequent attenuation of biosynthetic activity.

### 3.8. Identification of Flavonoid Biosynthesis Genes in P. grandiflorus Based on Transcriptome Analysis

**Figure 9:**
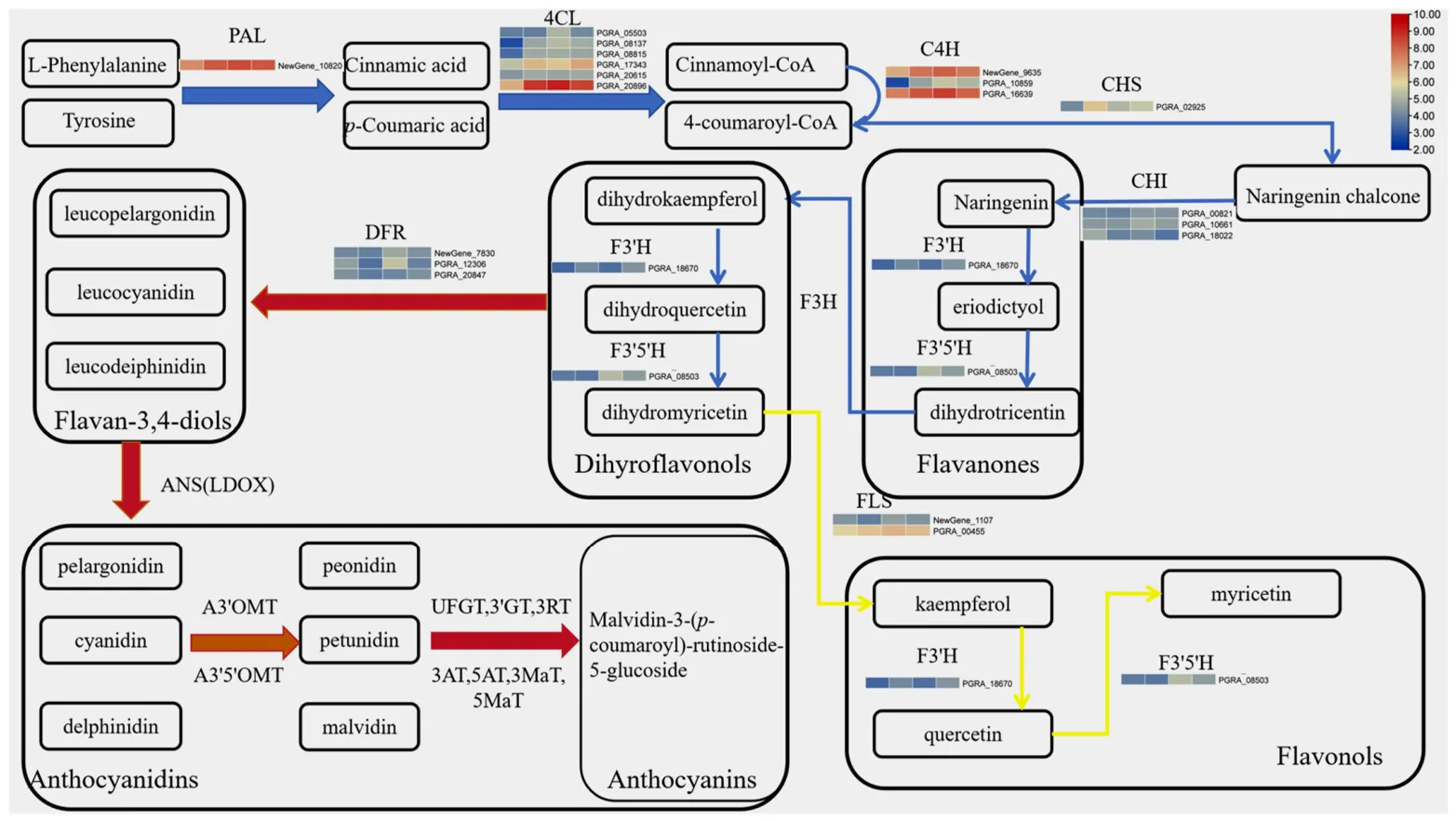
Schematic diagram of the biosynthetic pathway of flavonoid compounds in the root of *P. grandiflorus*. Blue and yellow arrows represent the early and late steps of flavonoid biosynthesis, respectively, while the red arrow indicates the biosynthetic pathway of anthocyanins. The abbreviations near the arrows represent the enzymes catalyzing the corresponding processes. The heat map near the arrows is used to characterize the expression levels of the flavonoid structural genes of *P. grandiflorus* at different times under MeJA treatment. PAL, phenylalanine ammonia-lyase; 4CL, hydroxycinnamic acid-CoA ligase; C4H, cinnamic acid 4-hydroxylase; CHS, chalcone synthase; CHI, chalcone isomerase; F3H, F3′H, and F3′5′H, flavonoid-3, -3′, and -3′5′-hydroxylases; FLS, flavonol synthase; DFR, dihydroflavonol-4-reductase; ANS/LDOX, anthocyanin synthase/bioflavonoid dioxygenase; A3′OMT and A3′5′OMT, anthocyanin 3′- and 3′5′-O-methyltransferases; UFGT, UDP-glucose: flavonoid-3-sugar glucosyltransferase; 3′GT, anthocyanin 3′-O-β-glucosyltransferase; 5GT, anthocyanin-5-O-glucosyltransferase; 3RT, UDP-mannose: anthocyanin-3-sugar glucosyltransferase; 3AT and 5AT, 3- and 5-aromatic acyl transferases; 3MaT, malonyl-CoA: anthocyanin-3-O-glucoside-6-O-malonyltransferase; 5MaT, malonyl-CoA: anthocyanin-5-O-glucoside-6-O-malonyltransferase.

Based on transcriptome-derived annotations of structural genes associated with flavonoid biosynthesis in *P. grandiflorus* whole roots, a comprehensive schematic representation of the core flavonoid pathway was reconstructed (Figure 9). The pathway encompasses 22 functionally annotated structural genes. Of these, 12 genes exhibited maximal transcript abundance at 12 h post- MeJA treatment. Critically, all 22 genes displayed a consistent up-regulation trajectory during the active phase of flavonoid accumulation, with expression levels significantly elevated relative to the untreated control (T0) at one or more time points, thereby supporting coordinated transcriptional activation of the entire pathway in response to MeJA elicitation.

### 3.9. The Effect of MeJA on the Expression of PgbHLH Genes in the Whole Roots of P. grandiflorus

**Figure 10:**
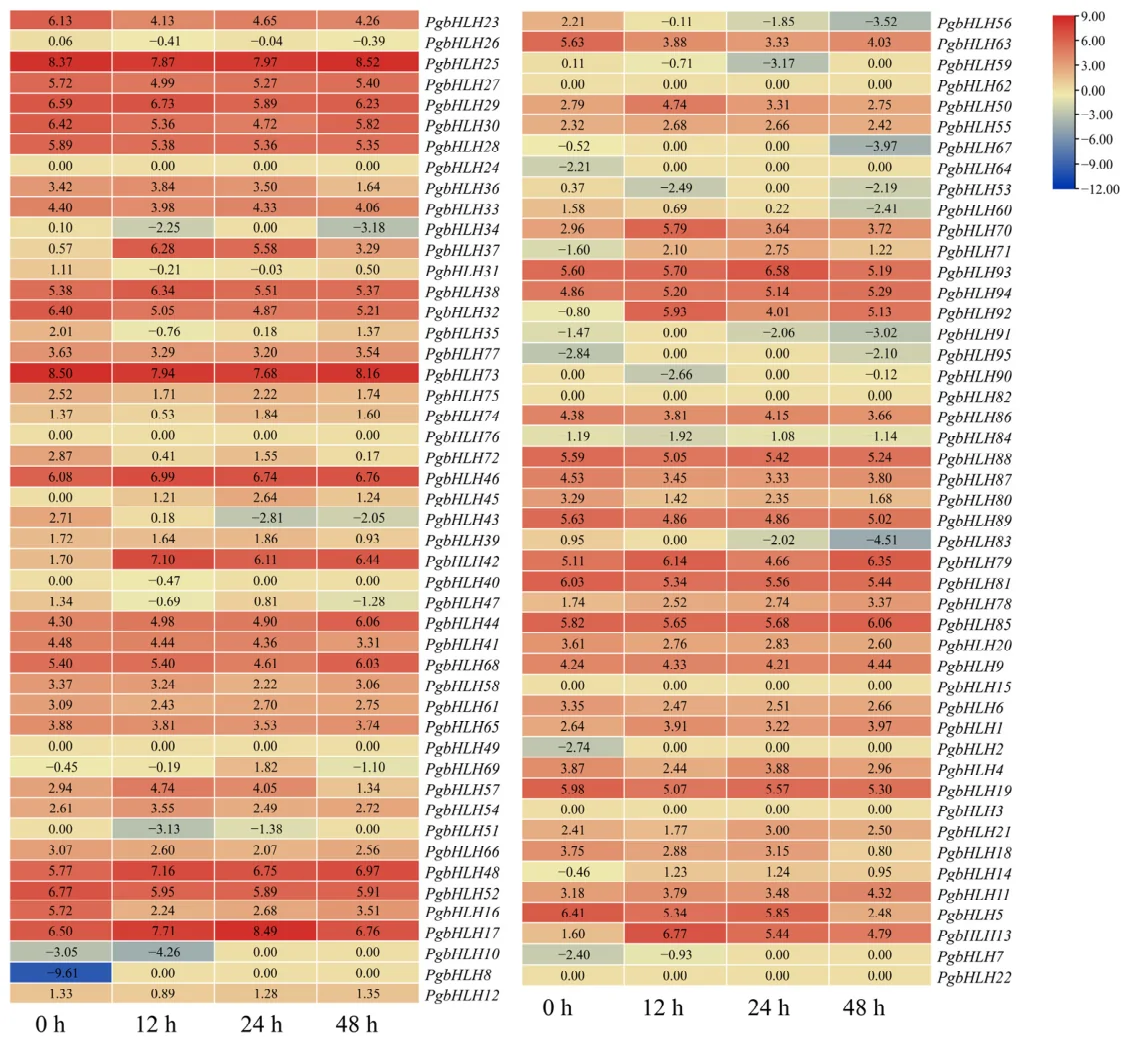
The heatmap of *bHLH* gene expression in the whole roots of *P. grandiflorus* at different time points under MeJA treatment.

To elucidate the impact of MeJA on *bHLH* gene expression in the whole roots of *P. grandiflorus*, the expression data of the *bHLH* gene after treatment with MeJA for 0 h, 12 h, 24 h and 48 h were screened from transcriptome data. As depicted in Figure 10, 7.36% of the genes exhibited no expression, while 21.05% of the genes increased post-MeJA treatment, with most genes reaching peak expression at the 12 h mark. Notably, *PgbHLH13*, *PgbHLH37*, *PgbHLH42*, *PgbHLH48*, *PgbHLH70*, and *PgbHLH92* demonstrated the most significant up-regulation. The expression patterns of *PgbHLH37*, *PgbHLH42*, and *PgbHLH92* initially increased and then decreased, with a marked elevation at 12 h followed by a substantial decline at 48 h. Conversely, 71.57% of the genes showed decreased expression after MeJA treatment, with 19.05% of these genes experiencing a reduction to zero expression. These findings indicated that MeJA might exert a dual regulatory effect on *bHLH* gene expression in the whole roots of *P. grandiflorus*: while the expression of most genes was suppressed under MeJA treatment, a subset of genes with increased expression achieved optimal induction at 12 h. MeJA, a classical signaling molecule involved in the regulation of plant flavonoid secondary metabolism, specifically induced the expression of key genes in the flavonoid biosynthesis pathway. Notably, the expression levels of *PgbHLH13*, *PgbHLH37*, *PgbHLH42*, *PgbHLH48*, *PgbHLH70*, and *PgbHLH92* genes were significantly up-regulated following MeJA treatment. Given the specific regulatory role of MeJA in the flavonoid biosynthesis pathway, it is plausible that these *PgbHLH* family genes may participate in the regulation of this pathway.

### 3.10. Prediction of Interaction Between the Members of the PgbHLH TF Family and Flavonoid Biosynthesis Genes and Related Expression Analysis

**Figure 11:**
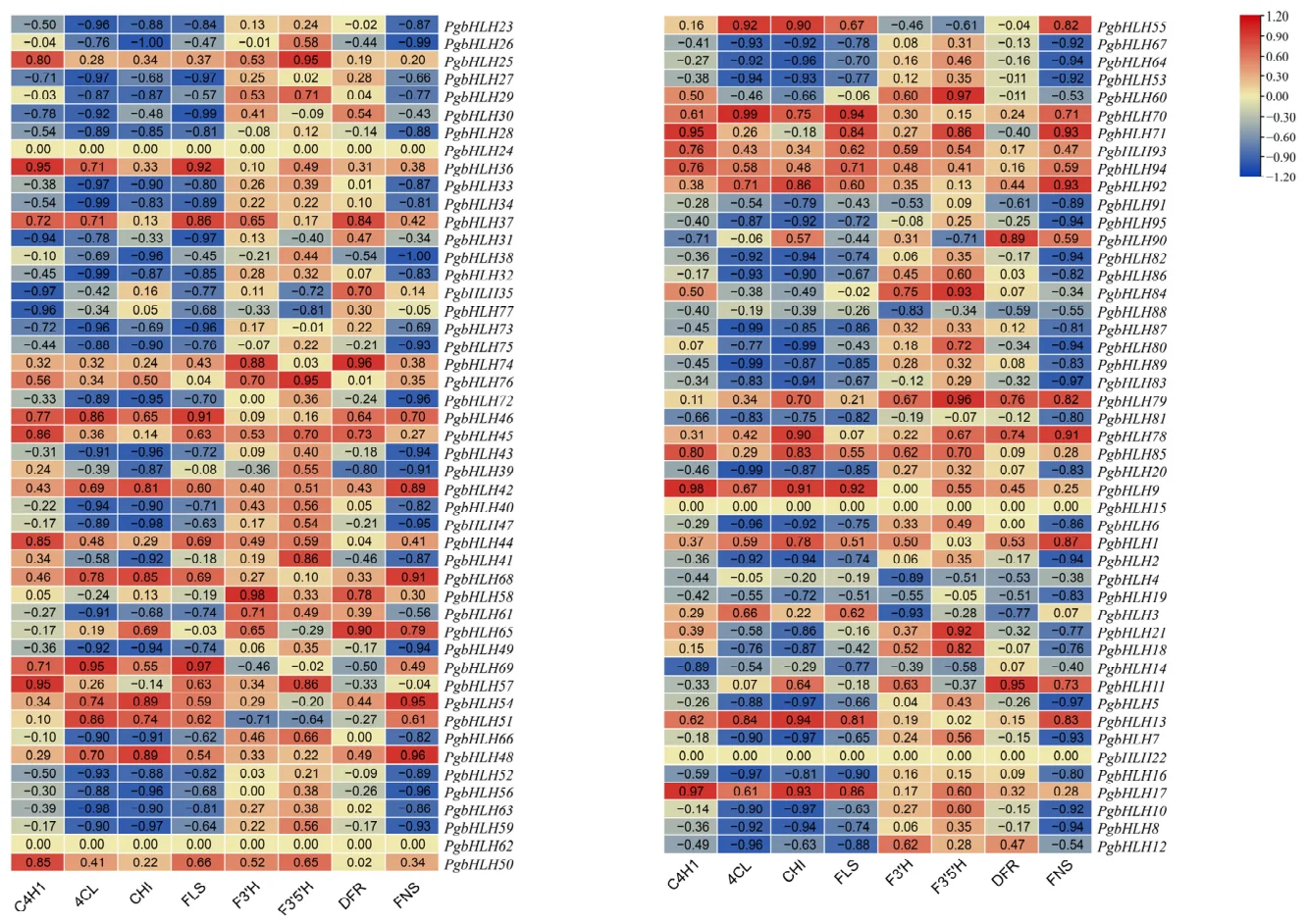
Heat map of correlation between *bHLH* gene and flavonoid synthesis structure gene in *P. grandiflorus.*

Eight structural genes related to flavonoid biosynthesis were selected based on the sequencing data: cinnamic acid-4-hydroxylase (C4H), 4-caffeoyl-CoA ligase (4CL), chalcone isomerase (CHI), flavonol synthase (FLS), flavonoid 3′-hydroxylase (F3′H), flavonoid 3′,5′-hydroxylase (F3′5′H), dihydroflavonol reductase (DFR), and flavonoid synthase (FNS). To elucidate the regulatory relationship between *PgbHLH* genes and flavonoid biosynthesis-related structural genes, this study first analyzed the expression profiles of flavonoid biosynthesis structural genes under MeJA treatment, followed by a correlation analysis with the expression levels of *PgbHLH* genes. The significant correlation results were visualized using TBtools software in the form of a heatmap (Figure 11). The analysis revealed that *PgbHLH22*, *PgbHLH24*, and *PgbHLH62* exhibited no significant correlation with any of the flavonoid biosynthesis-related structural genes. A total of 23 *PgbHLH* genes showed significant positive correlations with 8 flavonoid biosynthesis structural genes. Among these structural genes, F3′H and F3′5′H displayed the highest number of positive correlations, showing significant associations with 69 and 73 *PgbHLH* genes, respectively.

**Figure 12:**
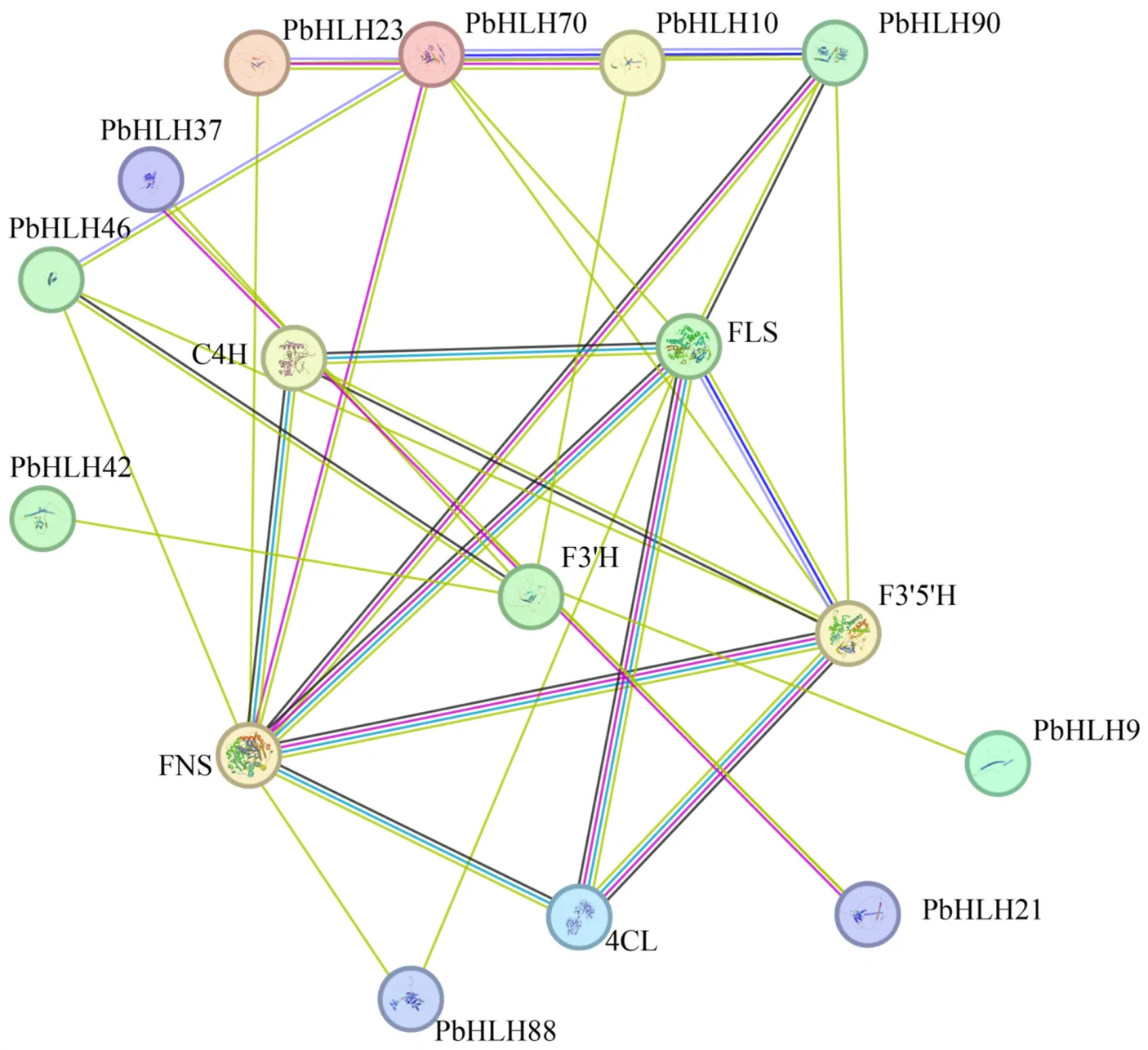
Predicted protein interaction network between bHLH genes and flavonoid biosynthetic structural proteins in *P. grandiflorus*. The color and quantity of the lines indicate the basis for the interaction between proteins. Interactions between 95 PgbHLH proteins (homologous to Arabidopsis bHLHs) and six flavonoid structural proteins (C4H, FNS, F3′H, F3′5′H, 4CL, FLS) were predicted using the STRING database. Nodes represent proteins, and connecting lines indicate predicted interactions, with line color and thickness reflecting evidence strength. Ten PgbHLH proteins (e.g., PgbHLH70, PgbHLH37, PgbHLH42) were predicted to interact with these structural proteins. Specifically, PgbHLH70 is predicted to interact with F3′5′H, FLS, and FNS; PgbHLH37 with C4H; and PgbHLH42 with F3′H, suggesting that these bHLH factors may be candidate participants in flavonoid regulation. It should be noted that these are computational predictions based on homology and require experimental validation.

The interaction between proteins is helpful to understand the regulatory pathways and potential mechanisms of genes. In order to screen the PgbHLH TF family members involved in the regulation of flavonoid synthesis in *P. grandiflorus*, a correlation analysis was conducted between the AtbHLH protein homologous to 95 PgbHLH proteins and the screened flavonoid synthesis-related proteins, and only the proteins with interaction relationship (C4H, FNS, F3′H, F3′5′H, 4CL, FLS) were retained. The results are shown in Figure 12. There were interaction relationships among the flavonoid synthesis proteins. Additionally, the 10 PgbHLH proteins were computationally predicted to interact with the 6 structural proteins related to flavonoid synthesis, and there was a widespread interaction among the flavonoid synthesis-related proteins. Among them, PgbHLH90 and PgbHLH70 were predicted to interact with F3′5′H, FLS, and FNS respectively, PgbHLH46 was predicted to interact with F3′5′H, F3′H, and FNS, and PgbHLH88 interacted with FNS and FLS. The remaining PgbHLH proteins interacted with only one flavonoid synthesis-related protein: PgbHLH10 interacted with F3′H, PgbHLH23 with FNS, PgbHLH37 with C4H, PgbHLH45 with F3′H, PgbHLH21 with C4H, and PgbHLH9 with F3′H. Based on the above protein interaction network results, it can be preliminarily speculated that the members of the PgbHLH family have putative interactions with the structural proteins involved in flavonoid synthesis and may participate in the regulation process of the flavonoid synthesis pathway in *P. grandiflorus* through this interaction mechanism. Through further analysis based on the correlation heatmap, candidate regulatory genes were identified according to three key criteria: (1) the expression levels were significantly up-regulated following MeJA treatment; (2) protein interaction predictions suggest that they can putatively associate with F3′5′H/FLS/FNS, C4H, and F3′H; and (3) expression correlation analysis revealed correlation coefficients greater than 0.9 between these genes and their corresponding interacting proteins, indicating a highly significant positive correlation. It is important to distinguish between transcriptional regulation of structural genes (inferred from expression correlations) and physical interaction with biosynthetic enzymes (predicted from homology-based STRING analysis). The present analysis does not demonstrate either process definitively; rather, it provides correlative and predictive evidence that requires experimental validation. Based on dual evidence of “expression co-regulation and predicted protein association,” it is hypothesized that the three *PgbHLH* genes (*PgbHLH37*, *PgbHLH42*, and *PgbHLH70*) would be likely to serve as candidate regulators in the flavonoid biosynthesis pathway in *P. grandiflorus*. In contrast, other *PgbHLH* genes (e.g., *PgbHLH9* and *PgbHLH21*) were considered potentially weakly regulated candidates, as they do not satisfy all three aforementioned criteria. Therefore, their regulatory influence in the flavonoid biosynthesis pathway may be minimal or less significant. The STRING-based predictions suggest potential physical associations, but these remain to be validated. Similarly, the expression correlations indicate co-expression, not direct transcriptional regulation. Therefore, all regulatory inferences drawn from these analyses are correlative and predictive, and should be considered hypotheses for future experimental testing.

### 3.11. Co-Expression Network of bHLH Genes in P. grandiflorus

**Figure 13:**
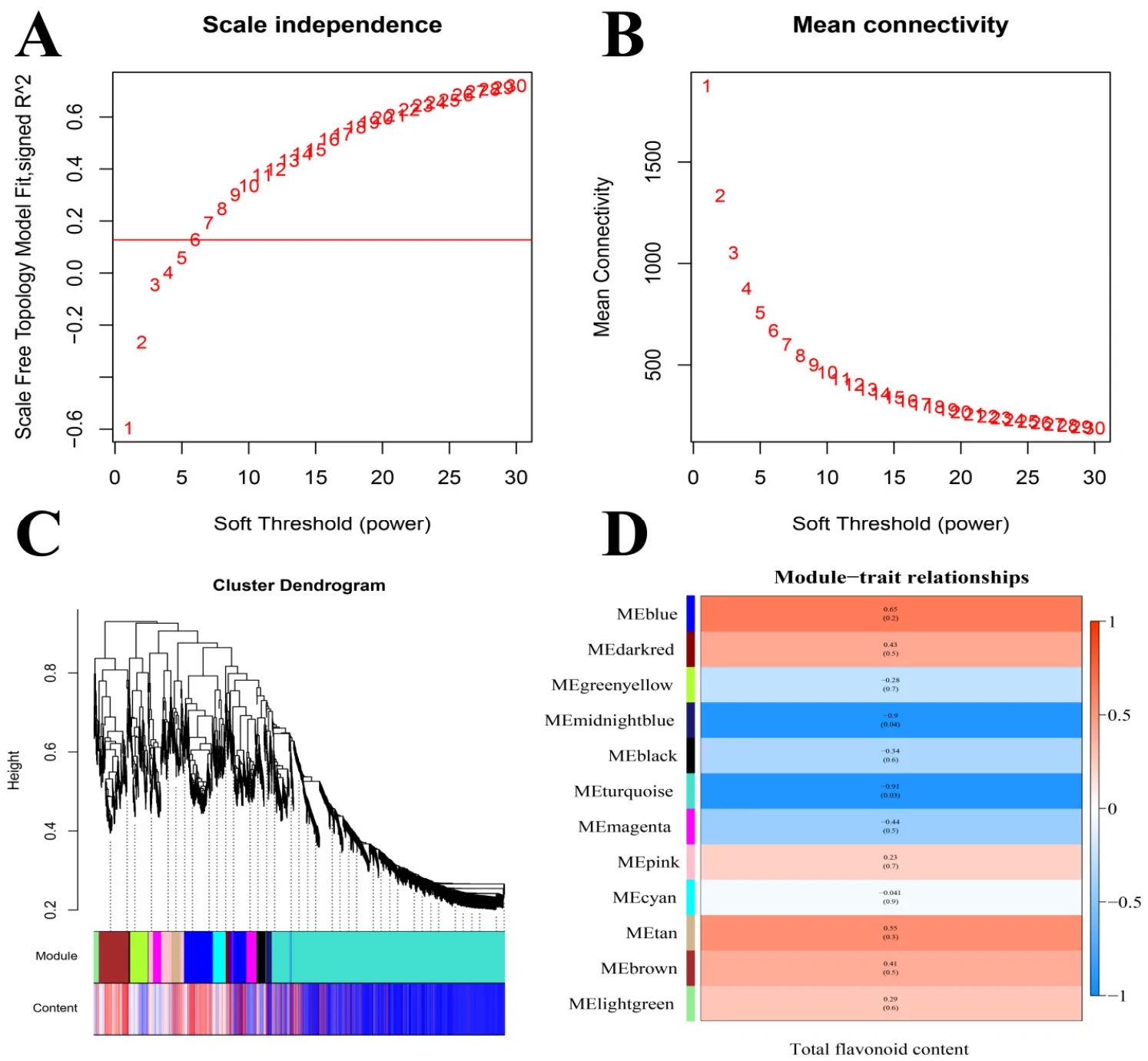
WGCNA of transcriptome and flavonoid content. (**A**,**B**) Analysis of network topology for various values of soft thresholding (power) and (**C**) construction of co-expression module. The same color represents the same module. If the module features genes between two different modules that are similar, they will be merged automatically. (**D**) Correlation heatmap between flavonoid content and 12 gene modules. The y-axis represents each module. The x-axis represents flavonoid content. Red and blue colors indicate up-regulated and down-regulated transcripts, respectively.

To investigate the correlation between the gene network and flavonoid content in *P. grandiflorus* and further refine the screening scope of upstream regulatory genes involved in flavonoid synthesis, a weighted gene co-expression network was constructed for the whole roots of *P. grandiflorus*. Ultimately, 12 modules (Figure 13D) comprising 2968 genes were identified from the clustering dendrogram. Each module contained a varying number of genes, with the “turquoise” module having the largest number (1755 genes) and the “lightgreen” module having the smallest (22 genes), averaging 247 genes per module. By establishing a co-expression network associated with the traits, the module exhibiting the highest correlation with the target traits was selected. As depicted in Figure 13D, six of the twelve modules were positively correlated with flavonoid synthesis, while the other six were negatively correlated. The MEblue and MEturquoise modules demonstrated the highest positive and negative correlations, respectively, with total flavonoid content, containing 323 and 1755 genes, respectively. *PgbHLH42* (kME = 0.9380) was localized within the MEblue module, whereas *PgbHLH37* (kME = 0.9584) and *PgbHLH70* (kME = 0.9461) were localized within the MEbrown and MEtan modules, respectively. A comprehensive analysis of these findings further suggests that *PgbHLH70*, *PgbHLH37*, and *PgbHLH42* are co-expressed with flavonoid-related modules and thus represent candidate regulatory genes, but WGCNA identifies co-expression patterns and correlations with phenotypic traits; however, it does not imply causation or direction of regulation. The WGCNA was based on 12 samples (three biological replicates × four time points), which is a modest sample size for robust network inference; therefore, the identified modules and hub genes should be considered exploratory and require validation with larger datasets.

### 3.12. Subcellular Localization of Candidate TFs bHLH37, bHLH42, and bHLH70 Related to Flavonoid Synthesis in P. grandiflorus

**Figure 14:**
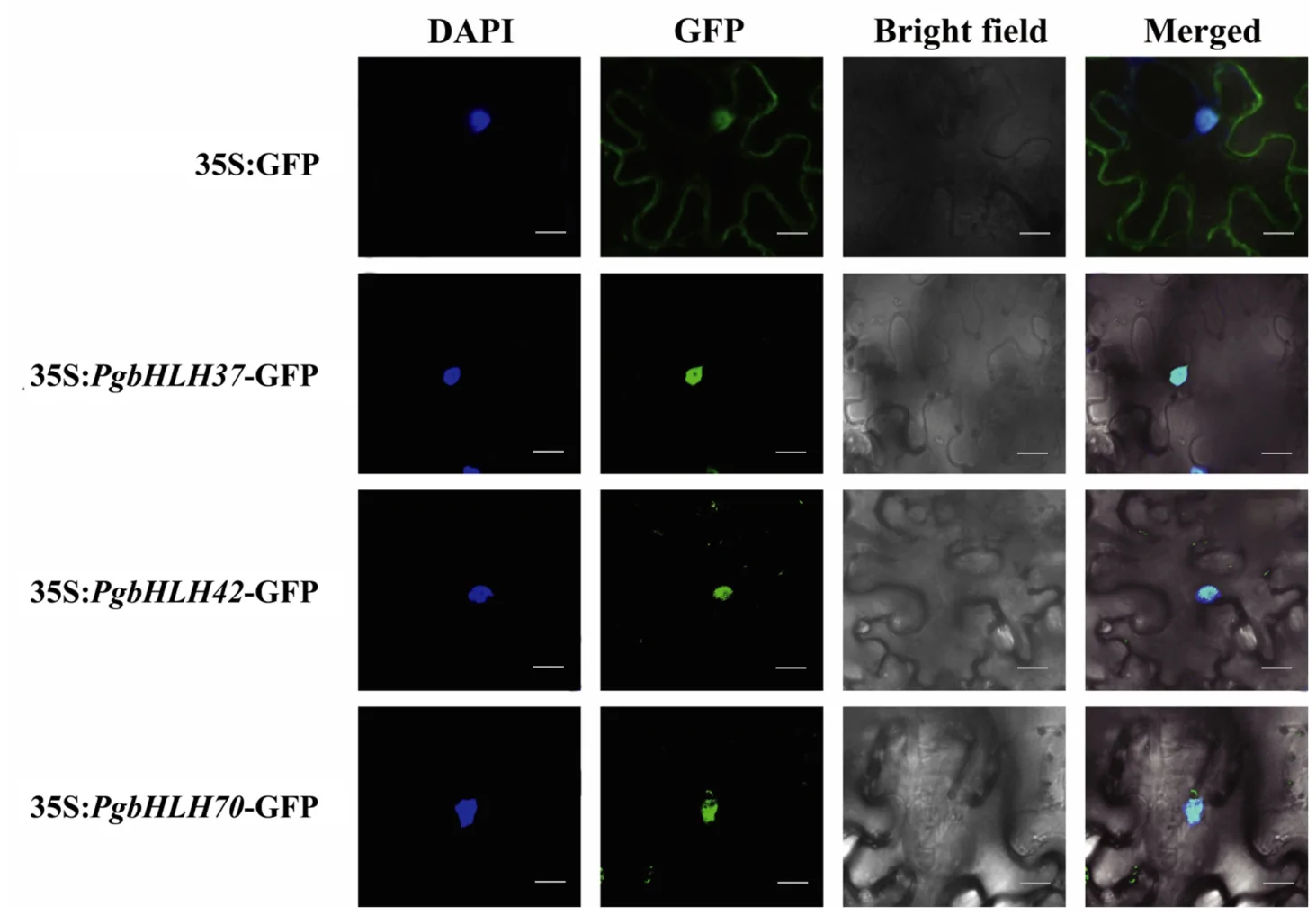
Subcellular localization of bHLH37, bHLH42, and bHLH70 proteins in *N. benthamiana* leaf epidermal pavement cells. Fusion constructs PgbHLH37-GFP, PgbHLH42-GFP, and PgbHLH70-GFP, along with the empty vector 35S:GFP (control), were transiently expressed in *N. benthamiana* epidermal cells. Nuclei were stained with DAPI (blue), and GFP fluorescence (green) indicates protein distribution. The 35S:GFP control showed fluorescence throughout the cell (cytoplasm and nucleus), whereas all three fusion proteins exhibited GFP signals exclusively overlapping with DAPI-stained nuclei, confirming nuclear localization of PgbHLH37, PgbHLH42, and PgbHLH70, consistent with their roles as transcriptional regulators. Scale bar = 20 μm. However, the heterologous expression system used here is a limitation; the results should be interpreted with caution.

To further investigate the subcellular localization of the candidate TFs PgbHLH37, PgbHLH42, and PgbHLH70, transient expression of PgbHLH37-GFP, PgbHLH42-GFP, and PgbHLH70-GFP fusion proteins was analyzed in tobacco leaves. The results revealed that GFP signals were detected in both the nuclei and cell membranes of epidermal cells in *N. benthamiana* leaves expressing 35S:GFP alone. In contrast, GFP signals were localized exclusively to the nuclei in epidermal cells expressing 35S:PgbHLH37-GFP, 35S:PgbHLH42-GFP, or 35S:PgbHLH70-GFP (Figure 14). These findings indicated that PgbHLH37, PgbHLH42, and PgbHLH70 were nuclear-localized proteins and likely to function as transcriptional regulators. Nevertheless, the localization data obtained via a heterologous transient expression system are inherently subject to methodological constraints. Although these results indicate nuclear localization, the heterologous transient expression system may not fully reflect the native behavior in *P. grandiflorus*; future studies using homologous systems are warranted.

**Figure 15:**
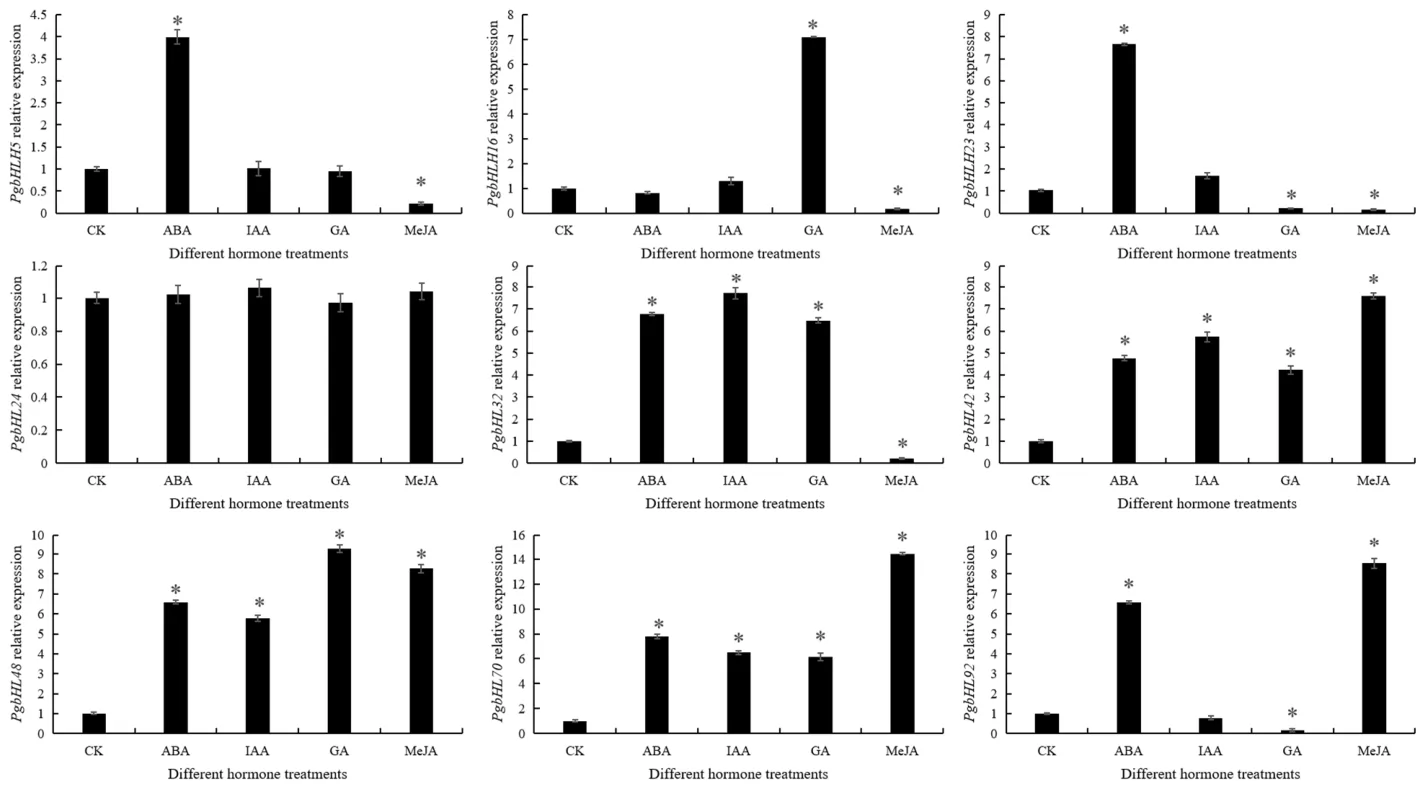
Expression pattern analysis of *PgbHLH* gene family members under four hormone treatments (100 μmol·L^−1^ IAA, GA, ABA, or MeJA). Data are presented as mean ± SD (*n* = 3 biological replicates, each with three technical replicates). Asterisks indicate significant differences between treated and control samples as determined by Student’s *t*-test (two-tailed, unequal variance): * *p* < 0.05.

Based on transcriptome data under MeJA treatment, correlation coefficients were calculated between 95 *PgbHLH* genes and eight key flavonoid structural genes (*C4H*, *4CL*, *CH*I, *FLS*, *F3′H*, *F3′5′H*, *DFR*, and *FNS*). The color scale from blue to red represents negative to positive correlations. A total of 23 *PgbHLH* genes showed significant positive correlations (coefficient > 0.9) with at least one structural gene; notably, *F3′H* and *F3′5′H* were significantly correlated with 69 and 73 *PgbHLH* genes, respectively, suggesting widespread involvement of these *bHLH* members in transcriptional regulation of the flavonoid pathway. To investigate the response of *PgbHLH* genes to exogenous hormones and stresses, four *PgbHLH* genes with significantly down-regulated expression, one gene with no detectable expression, and four genes with dramatically up-regulated expression were selected for RT-qPCR analysis based on RNA-seq data. As shown in Figure 15, the expression levels of *PgbHLH*42, *PgbHLH*48, and *PgbHLH*70 increased under treatment with four exogenous hormones, whereas *PgbHLH*24 exhibited no response to these hormones. Under MeJA treatment, the expression levels of *PgbHLH*5, *PgbHLH*16, *PgbHLH*23, and *PgbHLH*32 were noticeably down-regulated, while *PgbHLH*24 remained almost unchanged. The relative expression levels of other genes increased, with *PgbHLH*70 showing the highest up-regulation, reaching 14.42 times that of the control. Furthermore, the trend in gene expression under MeJA treatment was largely consistent with the RNA-seq data. Most genes were sensitive to ABA, except for *PgbHLH*16 and *PgbHLH*24, whose relative expression levels showed no significant changes. Under GA treatment, the expression of *PgbHLH*23 and *PgbHLH*92 was down-regulated, *PgbHLH*5 and *PgbHLH*24 remained largely unchanged, and other genes increased significantly, with *PgbHLH*48 showing the greatest increase, 9.27 times that of the control. The expression levels of *PgbHLH*32, *PgbHLH*42, *PgbHLH*48, and *PgbHLH70* showed pronounced increases under IAA treatment, while the expression of the other genes remained stable.

### 3.13. Expression Pattern of PgbHLH Genes Under Exogenous Hormones and Stress Treatments

To investigate the response of *PgbHLH* genes to exogenous hormones and stresses, four *PgbHLH* genes with significantly down-regulated expression, one gene with no detectable expression, and four genes with dramatically up-regulated expression were selected for RT-qPCR analysis based on RNA-seq data. These RT-qPCR experiments under multiple hormone and stress treatments were designed to test the responsiveness of candidate genes rather than to perform global transcriptome profiling. As shown in Figure 15, the expression levels of *PgbHLH*42, *PgbHLH*48, and *PgbHLH*70 increased under treatment with four exogenous hormones, whereas *PgbHLH*24 exhibited no response to these hormones. Under MeJA treatment, the expression levels of *PgbHLH*5, *PgbHLH*16, *PgbHLH*23, and *PgbHLH*32 were noticeably down-regulated, while *PgbHLH*24 remained almost unchanged. The relative expression levels of other genes increased, with *PgbHLH*70 showing the highest up-regulation, reaching 14.42 times that of the control. Furthermore, the trend in gene expression under MeJA treatment was largely consistent with the RNA-seq data. Most genes were sensitive to ABA, except for *PgbHLH*16 and *PgbHLH*24, whose relative expression levels showed no significant changes. Under GA treatment, the expression of *PgbHLH*23 and *PgbHLH*92 was down-regulated, *PgbHLH*5 and *PgbHLH*24 remained largely unchanged, and other genes increased significantly, with *PgbHLH*48 showing the greatest increase, 9.27 times that of the control. The expression levels of *PgbHLH*32, *PgbHLH*42, *PgbHLH*48, and *PgbHLH70* showed pronounced increases under IAA treatment, while the expression of the other genes remained stable.

**Figure 16:**
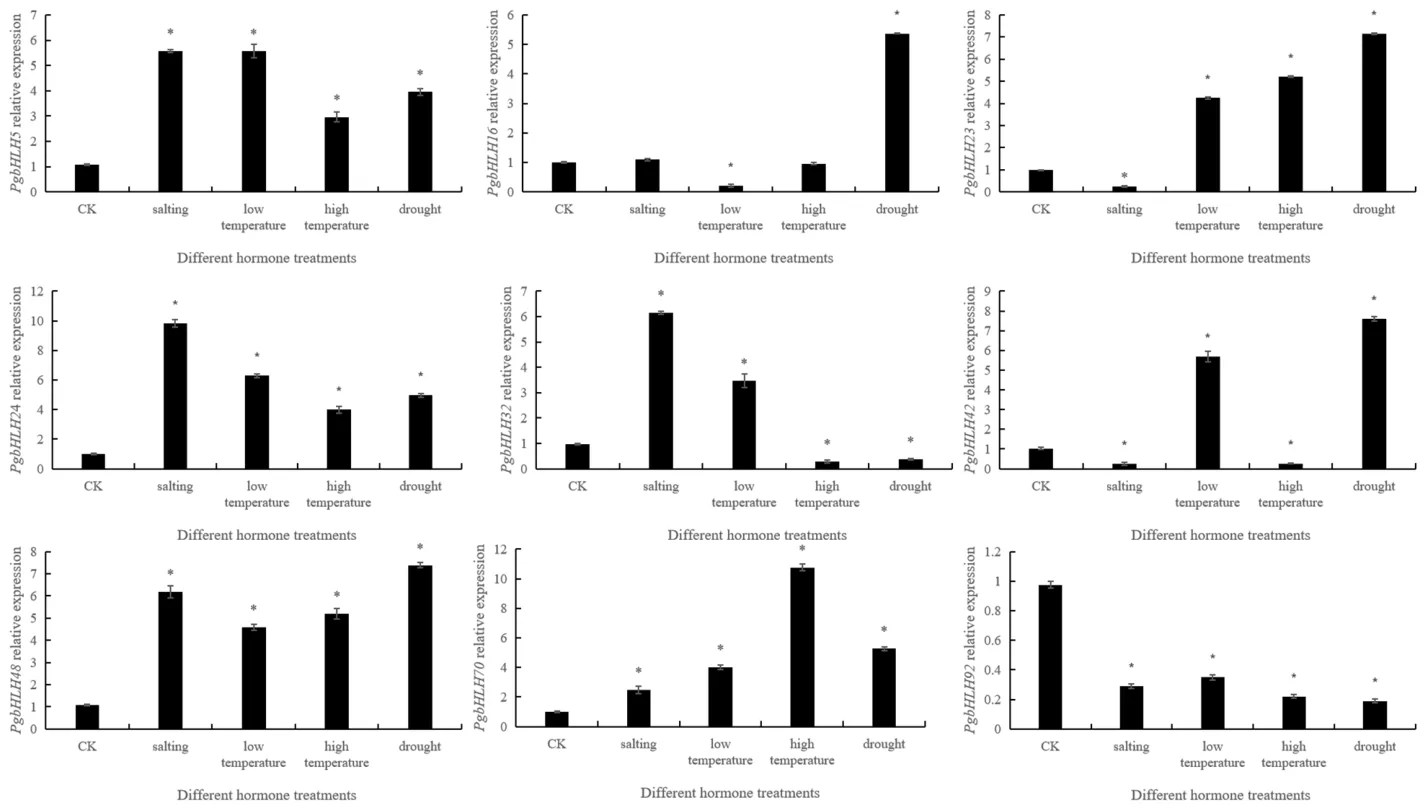
Expression pattern analysis of *PgbHLH* gene family members under four stress treatments (drought, salt, cold, and heat). Data are presented as mean ± SD (*n* = 3 biological replicates, each with three technical replicates). Asterisks indicate significant differences between treated and control samples as determined by Student’s *t*-test (two-tailed, unequal variance): * *p* < 0.05.

The results from the four stress treatments (as shown in Figure 16) revealed that under salt stress, the expression levels of *PgbHLH*5, *PgbHLH*24, *PgbHLH*32, and *PgbHLH*48 were significantly up-regulated, with *PgbHLH*24 showing a 9.82-fold increase compared to the control. Conversely, the expression levels of *PgbHLH*23, *PgbHLH*42, and *PgbHLH*92 were remarkably down-regulated, while the relative expression levels of the remaining genes did not exhibit obvious changes. Under low-temperature stress, the relative expression levels of *PgbHLH*16 and *PgbHLH*92 were noticeably decreased, whereas the expression levels of the other genes were substantially elevated. Under high-temperature stress, the relative expression levels of *PgbHLH*5, *PgbHLH*23, *PgbHLH*24, *PgbHLH*48, and *PgbHLH*70 were up-regulated; the relative expression level of *PgbHLH*16 remained relatively unchanged, while those of the other genes were down-regulated. Notably, *PgbHLH*70 exhibited the highest up-regulation, with a 10.76-fold increase compared to the control. Under drought stress, the relative expression levels of *PgbHLH*32 and *PgbHLH*92 were down-regulated, whereas those of the other genes were up-regulated, with *PgbHLH*23 and *PgbHLH*42 showing the highest increases of 7.15-fold and 7.59-fold, respectively.

## 4. Discussion

bHLH TFs are of paramount importance in a multitude of processes associated with plant growth, metabolism, and responses to abiotic stress [37]. To date, the number of identified bHLH TFs in various plant species is as follows: 208 in maize [5], 161 in *Arabidopsis* [7], 183 in rice [8], and 100 in rose [38]. In the present study, 95 *PgbHLH* genes were screened and identified via bioinformatics analysis. To explore the evolutionary relationship between *Arabidopsis* and *PgbHLH* TFs, all *bHLH* members of these two species were classified into 25 subgroups. Among them, 23 subgroups contained representatives from both *Arabidopsis* and *P. grandiflorus*, suggesting a high degree of homology between the two species. Previous research has indicated that members within the same subgroup may share similar biological functions. For example, members of the VII family in *Arabidopsis* play a pivotal role in light signal regulation, and genes such as *AtbHLH108* and *AtbHLH92* in the XII subfamily of *Arabidopsis* have been recognized as key genes for anthocyanin synthesis [39]. Consequently, the regulatory functions of *PgbHLH* in *P. grandiflorus* can be deduced based on the functions of *bHLH* genes in *Arabidopsis* [40,41]. Furthermore, it was observed that the number of *AtbHLH* genes in multiple subfamilies exceeded that of *PgbHLH* genes. This disparity might be attributed to gene duplication and loss events during the evolutionary process [42]. By mapping the chromosomal location of the genes, it was found that *PgbHLH* genes were distributed across nine chromosomes. Specifically, chromosome 5 harbored the highest number of *PgbHLH* genes, while chromosome 9 had the lowest number, indicating an uneven distribution of genes on different chromosomes during evolution. Notably, genes on chromosomes 1 and 5 were closely spaced and clustered, which could potentially be the result of tandem gene duplication [43]. The uneven chromosomal distribution suggests that segmental duplications played a role in shaping the current genomic arrangement of *PgbHLH* genes. Synteny analysis demonstrated that gene duplication events occurred on seven chromosomes. Among the 36 gene duplications, 33 were segmental duplications. This finding suggests that both segmental and tandem duplications contribute to the expansion of the gene family, with segmental duplication playing a more prominent role [44]. Through Ka/Ks analysis of *PgbHLH* gene duplication events, it was determined that all Ka/Ks values were less than 1. This indicates that *PgbHLH* genes are under strong purifying selection pressure, preserving their functions and suggesting that they have not undergone significant functional divergence during evolution, thereby highlighting their strong conservation. We acknowledge that lineage-specific clades may exist but were not resolved due to the lack of a dedicated *P. grandiflorus*-specific classification. The predominance of segmental duplications suggests that genome-wide duplication events may have contributed to the expansion of the bHLH family in *P. grandiflorus*. Furthermore, purifying selection does not exclude the possibility of neofunctionalization; future studies on expression divergence are warranted.

Structural variations play a crucial role in gene evolution, with introns evolving in parallel with genomic changes within organisms. This evolutionary process is primarily reflected in the gain or loss, insertion, or deletion of introns and exons [43]. Gene structure analysis revealed that members of the same subfamily exhibit both similarities and distinctions, suggesting potential functional conservation within subfamilies. Notably, all three genes lacking introns were clustered within the XIX subfamily, supporting the reliability of subfamily classification. All *PgbHLH* genes encoded bHLH-related domains, and genes sharing similar domains were typically located in the same or adjacent subfamilies, implying possible functional similarity among neighboring subfamilies [45]. Comparative analysis of conserved motifs across different subfamilies further demonstrated that genes within the same subfamily possessed analogous motif patterns, reinforcing the hypothesis of shared functional attributes [46]. Furthermore, certain motifs exhibit subfamily-specific distribution patterns. For example, Motif 3 is exclusively present in subfamilies XX to XXVI of the *PgbHLH* gene family, whereas Motif 5 is uniquely identified in subfamily XII. The distinctiveness and conservation of these motifs offer important insights into the evolutionary classification and functional divergence of the *PgbHLH* gene family.

Cis-regulatory elements present in promoter regions significantly influence gene function [47]. Promoter prediction results indicated that *PgbHLH* genes contained numerous regulatory elements responsive to hormones, stress, and growth and developmental cues. These findings suggest that *PgbHLH* genes may be involved in various biological processes in *P. grandiflorus*, including growth, secondary metabolism, and responses to biotic and abiotic stresses—observations consistent with previous studies on bHLH TFs in maize [5], *Arabidopsis* [7], and rice [8]. Nevertheless, these cis-element predictions are computational and require experimental validation (e.g., promoter–reporter assays) to confirm their functional relevance. Additionally, 77.89% of *PgbHLH* genes harbored MYB-binding sites. Previous studies have demonstrated that plant bHLH TFs commonly interact with MYB proteins to form functional complexes. These complexes are involved in the direct or indirect regulation of downstream genes associated with plant secondary metabolism, and distinct MYB-bHLH complexes can mediate the regulation of diverse physiological processes [48,49]. Based on this evidence, it is hypothesized that the PgbHLH TF in *P. grandiflorus* may form a complex with the PgMYB protein to cooperatively participate in regulating multiple physiological processes in this species.

Research into the regulation of flavonoid biosynthesis by plant bHLH TFs has advanced steadily. Anthocyanins represent a class of flavonoid compounds, with their biosynthetic pathway forming a key branch of the broader flavonoid biosynthesis pathway. Studies have shown that in peonies, *PsbHLH1* positively regulates anthocyanin biosynthesis by directly binding to the promoters of dihydroflavonol 4-reductase (PsDFR) and anthocyanidin synthase (PsANS), thereby activating their transcription [17]. Similarly, in strawberries, the MYB-bHLH-WD40 protein complex plays a central role in regulating anthocyanin synthesis within the flavonoid biosynthesis pathway [50]. Protein interaction analysis serves as a critical approach for elucidating the molecular mechanisms underlying biological regulatory pathways. Based on sequencing data, this study initially identified eight core structural proteins involved in the flavonoid biosynthesis pathway, including C4H, 4CL, and CHI. Subsequently, 95 PgbHLH family proteins from *P. grandiflorus* root were selected for interaction prediction with the aforementioned structural proteins associated with flavonoid synthesis. It should be emphasized that transcription factors (such as bHLH proteins) regulate gene expression by binding to DNA and modulating transcription of structural genes, whereas biosynthetic enzymes (such as C4H, 4CL, and CHI) catalyze chemical reactions in the metabolic pathway. The STRING-based predictions described below suggest potential physical associations between PgbHLH proteins and flavonoid biosynthetic enzymes, but these are computational predictions and should not be confused with transcriptional regulation of structural genes. The results indicated that a total of 10 PgbHLH proteins were predicted to interact with six types of structural proteins involved in flavonoid biosynthesis. According to the functional correlation derived from protein interactions, it is hypothesized that these 10 PgbHLH proteins may participate in the regulation of the *P. grandiflorus* flavonoid biosynthesis pathway through putative interactions with key structural proteins. Wang et al. [51] identified *CebHLH13* and *CebHLH75* in orchids, which are known to play crucial roles in anthocyanin biosynthesis and belong to the same subfamily as *AtbHLH55* and *AtbHLH80*. In this study, *PgbHLH70* was also found to belong to the same subfamily. Given that genes within the same subfamily may share similar functional roles [52], it is proposed that *PgbHLH70* may be involved in the regulation of the flavonoid biosynthesis pathway. Furthermore, in terms of the dual verification framework of “expression co-regulation and direct protein interaction,” the interaction patterns of PgbHLH37 and PgbHLH42 were found to be consistent with that of PgbHLH70. Specifically, all three genes were computationally predicted to interact with key structural proteins in the flavonoid biosynthesis pathway, namely C4H and F3′H. Expression correlation analysis further revealed correlation coefficients exceeding 0.9 between these genes and their corresponding interacting proteins, indicating a highly significant positive correlation. Additionally, following MeJA treatment, the expression levels of these three genes were markedly up-regulated. Therefore, it is hypothesized that PgbHLH37, PgbHLH42, and PgbHLH70 may collectively participate in the regulation of the *P. grandiflorus* flavonoid biosynthesis pathway. However, the precise molecular mechanisms—such as whether these proteins regulate target protein activity, influence expression levels, or affect subcellular localization—remain to be further elucidated. On the basis of the schematic representation of the flavonoid biosynthesis pathway in *P. grandiflorus* root, multiple structural genes were involved in the regulatory network and exhibited positive regulatory effects. This study also involved subcellular localization analysis of the PgbHLH37, PgbHLH42, and PgbHLH70 proteins, which were potentially involved in regulating flavonoid biosynthesis. The results demonstrated that all three proteins were localized in the nucleus, suggesting that they possess transcriptional regulatory capabilities. However, it should be noted that these localization results were obtained using a heterologous transient expression system in *N. benthamiana*, which may not fully reflect the native behavior in *P. grandiflorus*; future studies using homologous systems are warranted.

WGCNA is a systems-level bioinformatic approach that enables the identification of functionally coherent gene modules strongly associated with specific phenotypic traits [53,54]. By integrating expression profiles across biological replicates and time points, WGCNA facilitates the detection of hub genes—those exhibiting high module membership (kME) and strong correlation with trait data—which serve as high-priority candidates for functional validation [55]. However, it should be emphasized that WGCNA identifies co-expression patterns and correlations with traits, not causation or direction of regulation. Furthermore, the WGCNA was based on only 12 samples (three biological replicates × four time points), which is a relatively small sample size for network inference; consequently, the identified modules and hub genes should be interpreted as exploratory. To test the hypothesis that bHLH TFs regulate flavonoid biosynthesis in *P. grandiflorus* whole roots, a scale-free co-expression network using normalized transcriptome data from all 12 RNA-seq libraries was constructed. Module–trait association analysis revealed two modules significantly correlated with total flavonoid content: MEblue (r = 0.65) and METurquoise (r = −0.91). However, because only total flavonoid content was measured, it remains unknown whether specific flavonoid classes (e.g., anthocyanins, flavonols, or flavones) are differentially regulated. Within MEblue, *PgbHLH42* exhibited the highest module membership (kME = 0.9380) and positive correlation with flavonoid accumulation, suggesting a potential transcriptional activator role. *PgbHLH37* (kME = 0.9584) and *PgbHLH70* (kME = 0.9461) were localized in the MEbrown and MEtan modules, respectively, and may exert a promoting effect on the flavonoid synthesis pathway. Therefore, based on the aforementioned discussion, it is hypothesized that *PgbHLH37*, *PgbHLH42*, and *PgbHLH70* may represent high-priority candidate regulators potentially associated with flavonoid synthesis in *P. grandiflorus*, but these hypotheses require experimental validation (e.g., overexpression, knockout, or protein–protein interaction assays).

MeJA is a derivative of jasmonic acid and serves as an important regulatory factor in plant growth and secondary metabolite biosynthesis. Previous studies have demonstrated that MeJA can induce the expression of certain bHLH TFs in *P. grandiflorus*, thereby promoting the accumulation of saponin compounds and regulating downstream structural genes [23,24]. According to sequencing data, following MeJA treatment, the expression levels of 71.57% of *PgbHLH* genes were down-regulated, whereas 22.05% of these genes exhibited an initial increase followed by a decrease over time, with the most pronounced induction effect observed at 12 h. This suggests that MeJA treatment enhances the expression of specific *PgbHLH* genes in *P. grandiflorus*, which aligns with previous findings. Based on RNA-seq data, four genes showing significantly reduced expression, one gene with no detectable expression, and four genes exhibiting dramatically increased expression were selected for further hormone treatment experiments. The results revealed a high degree of consistency between the gene expression patterns observed after MeJA treatment and those derived from RNA-seq analysis, thereby reinforcing the reliability of the RNA-seq dataset. Extensive research has confirmed the involvement of bHLH TFs in ABA-mediated responses to drought stress [56]. In this study, all *PgbHLH* genes except *PgbHLH16* and *PgbHLH24* were found to be significantly up-regulated under ABA treatment, indicating their potential role in ABA-regulated stress response pathways. Under GA treatment, five genes displayed significant up-regulation, which may be attributed to the presence of GA-responsive promoter elements such as the P-box and GAER-motif within their promoter regions. Similarly, four genes showed marked increases in expression under IAA treatment, and all of them contained AuxRR-core IAA response elements. These correlations suggest that the corresponding genes may respond to these hormones, but direct regulation by these cis-elements remains to be experimentally confirmed. Studies have indicated that this cis-acting promoter element plays a crucial role in mediating IAA responses [57]. Collectively, these findings suggest that different *PgbHLH* genes exhibit distinct hormonal response patterns or signaling pathways.

To assess the stress-responsive potential of candidate bHLH TFs, quantitative RT-qPCR was performed on root tissues of *P. grandiflorus* subjected to four abiotic *stress* treatments—drought, salt, cold, and heat. Expression analysis revealed that *PgbHLH5*, *PgbHLH24*, and *PgbHLH48* were consistently up-regulated across all stress conditions, indicating broad-spectrum abiotic stress responsiveness. In silico promoter analysis further identified conserved cis-regulatory elements—including low-temperature-responsive LTR motifs and drought-inducible MYB-binding sites (MBS)—within the 2 kb upstream regions of these three genes, supporting their functional involvement in multi-stress adaptation. This concordance between transcriptional induction and promoter architecture aligns with established roles of orthologous *bHLH* genes in other plant species [58,59]. Under cold stress specifically, *PgbHLH16* and *PgbHLH92* exhibited significant down-regulation, whereas all other assayed *PgbHLH* genes showed robust up-regulation. The prevalence of LTR elements in the promoters of the induced genes suggested that cold-responsive transcriptional activation is likely mediated—at least in part—through canonical cold-signaling pathways [60].

Phylogenetic analysis grouped *PgbHLH23*, *PgbHLH42*, and *PgbHLH16* within the same clade as *AtbHLH79* (a known positive regulator of drought tolerance in *A. thaliana*), while *PgbHLH48*, *PgbHLH32*, and *PgbHLH24* clustered with *AtbHLH34* (a characterized mediator of salt stress adaptation). Crucially, expression patterns mirrored evolutionary relationships: the *AtbHLH79-*associated subgroup displayed pronounced up-regulation under drought, and the *AtbHLH34-*associated subgroup responded strongly to salt stress—consistent with functional conservation across eudicots [61].

Notably, several additional P*gbHLH* genes—including *PgbHLH7, PgbHLH39,* and *PgbHLH85*—also exhibited significant induction under drought or salt stress despite lacking close phylogenetic ties to *AtbHLH79* or *AtbHLH34*, implying the existence of lineage-specific or convergent regulatory mechanisms. Collectively, the divergent yet condition-specific expression profiles across the PgbHLH family underscore functional diversification within this TF family, reflecting specialized roles in orchestrating distinct abiotic stress response programs in *P. grandiflorus*.

It is important to acknowledge several limitations of this study. First, the protein–protein interactions are computational predictions based on homology with Arabidopsis proteins and require experimental validation (e.g., Y2H, BiFC, Co-IP). Second, subcellular localization was performed in a heterologous system (*N. benthamiana*), which may not fully reflect native behavior in *P. grandiflorus*. Third, only total flavonoid content was measured; quantification of individual flavonoid classes (e.g., by LC-MS/MS) is needed to assess pathway-specific regulation. Fourth, transcriptome sequencing was conducted exclusively for MeJA-treated samples, while other hormone and stress treatments were used only for RT-qPCR validation. Fifth, WGCNA was based on only 12 samples, a modest sample size for robust network inference; thus, the identified modules and hub genes should be considered exploratory. Sixth, pooling roots from multiple individuals per replicate precludes assessment of inter-individual variability. Finally, all regulatory inferences are correlative and predictive; direct functional validation (e.g., overexpression, knockout, or protein–protein interaction assays) is required to confirm the roles of candidate genes.

## 5. Conclusions

In this study, a total of 95 bHLH transcription factor genes were systematically identified from the high-quality reference genome of *P. grandiflorus*. Phylogenetic analysis classified these PgbHLH proteins into 23 distinct subfamilies, which exhibited non-uniform chromosomal distribution across all nine chromosomes. Sequence analysis confirmed that all PgbHLH proteins harbor the canonical bHLH domain; however, substantial variation in exon–intron organization and protein length was observed, implying functional diversification within the family. Intraspecific synteny analysis revealed 36 segmental or tandem duplication events, and Ka/Ks ratio analysis indicated that all duplicated gene pairs exhibited Ka/Ks < 1—consistent with strong purifying selection and evolutionary constraint. Cis-regulatory element prediction in upstream promoter regions (2.0 kb upstream of transcription start sites) demonstrated an enrichment of cis-elements associated with phytohormone signaling (e.g., ABA, MeJA, IAA, GA) and abiotic stress responses; notably, several promoters also harbored binding motifs linked to flavonoid biosynthesis regulation (e.g., MYB, bZIP, and light-responsive elements implicated in phenylpropanoid pathway activation). Subcellular localization assays via transient expression of GFP-tagged constructs in *Nicotiana benthamiana* epidermal cells confirmed nuclear localization for PgbHLH37, PgbHLH42, and PgbHLH70. Transcriptome profiling revealed that MeJA treatment elicited complex, isoform-specific transcriptional responses in *P. grandiflorus* whole roots: while the majority of *PgbHLH* genes were significantly down-regulated, a subset exhibited transient up-regulation or biphasic expression dynamics. These patterns were robustly validated by RT-qPCR, confirming high technical reproducibility and biological relevance. Integrative analysis, including predicted protein–protein interaction networks, co-expression correlation with structural genes of the flavonoid pathway (e.g., CHS, F3H, DFR, ANS), and module membership in WGCNA-derived co-expression modules, identified 23 PgbHLH proteins with significant associations with flavonoid biosynthesis. Among these, PgbHLH37, PgbHLH42, and PgbHLH70 emerged as top-tier candidates, demonstrating convergent evidence across multiple analytical dimensions: strong co-expression with core flavonoid genes, predicted interaction potential with key enzymatic partners, and modular centrality in flavonoid-enriched co-expression networks. Collectively, these findings position PgbHLH37, PgbHLH42, and PgbHLH70 as high-priority candidate regulators coordinating flavonoid accumulation in *P. grandiflorus*. However, it should be emphasized that the protein–protein interactions presented in this study are computational predictions and lack experimental validation (e.g., Y2H, BiFC, Co-IP); therefore, these interactions should be considered as hypotheses for future testing. Despite these limitations, our integrative genomic, transcriptomic, and network-based analyses provide a foundational framework for understanding bHLH-mediated flavonoid metabolism in *P. grandiflorus* and prioritize three high-confidence candidate regulators (PgbHLH37, PgbHLH42, and PgbHLH70) for future functional studies.

## Figures and Tables

**Table 1:** Primers for fluorescence quantification of *PgbHLH* gene.

Gene Name	Forward Primer (5′–3′)	Reverse Primer (5′–3′)
*PgbHLH5*	TCAGAAACTTGAGATTGGTGA	TTTGTAGGGACTGGACATAG
*PgbHLH16*	CGCTGAGAGGGTTAGGAGAG	TGGATAAGGATGCGAGGAGT
*PgbHLH23*	CTCCACATAACTCTTACCCA	ACTCAATCTTTTACGACCAT
*PgbHLH24*	TAGCCGATGTGGAGGTGAGG	AATGGTGGTGATGTGGTGTGA
*PgbHLH32*	TTATCAACTGCTCGTCCACT	TTTCTCCACCTTTAGCCTCA
*PgbHLH42*	GGTGGTAATGTCGCTGGCTC	TTCTGTTATGGCTTTGGGCT
*PgbHLH48*	AGAATGAACTGCGTGACGAGAA	TAACCCATGAATGGCATGAGC
*PgbHLH70*	GAGATTGAAAGGCAGCGTAG	GATTGGATTGATTGGAGTAA
*PgbHLH92*	GCTGATATTGGATTGGTCGG	CGTAATTGTGCTTTGGTCGT
*PgActin*	CCGTGGAGCCAAGGGTTG	GGAGCACCCAAGCTTGCG

## Data Availability

The datasets used and/or analyzed during the current study are available from the corresponding author on reasonable request.

## Supplementary Materials

The following supporting information can be downloaded at: https://www.mdpi.com/article/10.3390/genes17091144/s1. Table S1: Physicochemical properties of the *PgbHLH* gene family members in *Platycodon grandiflorus*. Table S2: Ka/Ks values of duplicated *PgbHLH* gene pairs.
