# Supplementary material for: Genome-Wide Identification of bHLH Transcription Factors and Screening of Candidate Regulators Associated with Flavonoid Biosynthesis in Platycodon grandiflorus Under Methyl Jasmonate Treatment
Source: Genes (Basel). 2026 Sep 18;17(9):1144. doi: 10.3390/genes17091144 (PMC13606143; doi:10.3390/genes17091144)
Supplement: Supplementary file 1 [file genes-17-01144-s001.zip › genes-4521581-supplementary.pdf]

Table S1. Physicochemical properties of the *PgbHLH* gene family members in *Platycodon grandiflorus*.

Gene name	Accession number	Number of Amino Acid	Molecular Weight	Theoretical pI	Instability Index	Aliphatic Index	Grand Average of Hydropathicity	Subcellular localization
PgbHLH1	GWHPARYT003933	397	43572.39	6.27	49.04	69.27	-0.62	nucl
PgbHLH2	GWHPARYT004205	168	19467.92	5.45	54.04	75.89	-0.70	nucl
PgbHLH3	GWHPARYT004223	604	68456.53	5.65	37.33	73.25	-0.65	nucl
PgbHLH4	GWHPARYT004355	510	55647.9	5.91	49.54	70.55	-0.62	nucl
PgbHLH5	GWHPARYT004367	366	40496.55	6.8	50.67	74.07	-0.53	nucl
PgbHLH6	GWHPARYT004700	274	29941.31	5.42	49.19	65.18	-0.71	nucl
PgbHLH7	GWHPARYT004757	94	10620.89	9.5	76.57	88.19	-0.76	nucl
PgbHLH8	GWHPARYT004761	129	14431.44	8.41	57.76	91.47	-0.46	nucl
PgbHLH9	GWHPARYT004766	239	26566.25	5.11	56.68	84.44	-0.54	nucl
PgbHLH10	GWHPARYT004842	394	43547.33	9.23	62.93	60.96	-0.63	nucl
PgbHLH11	GWHPARYT004930	598	65948.05	6.92	64.97	65.07	-0.80	nucl
PgbHLH12	GWHPARYT005047	441	48380.94	6.99	58.71	62.38	-0.65	nucl
PgbHLH13	GWHPARYT005249	256	28250.24	8.65	58.8	63.24	-0.49	nucl
PgbHLH14	GWHPARYT005260	306	33608.4	5.1	68.34	63.56	-0.67	nucl
PgbHLH15	GWHPARYT005353	373	41921.2	4.87	52.67	61.21	-0.87	nucl
PgbHLH16	GWHPARYT005402	408	44712.71	6.16	42.93	62.4	-0.67	nucl
PgbHLH17	GWHPARYT005453	347	39254.37	4.79	59.18	76.69	-0.46	nucl
PgbHLH18	GWHPARYT005575	322	35939.19	5.55	62.88	76.02	-0.52	nucl
PgbHLH19	GWHPARYT006076	333	36703.71	5.89	55.97	62.1	-0.84	nucl
PgbHLH20	GWHPARYT006262	456	50966.27	5.28	54.66	72.26	-0.49	nucl
PgbHLH21	GWHPARYT006449	331	37035.82	6.21	66.58	82.24	-0.60	nucl
PgbHLH22	GWHPARYT006549	302	34525.65	5.84	51.86	70.07	-0.65	nucl
PgbHLH23	GWHPARYT019017	570	62243.82	8.97	46.4	61.47	-0.69	nucl
PgbHLH24	GWHPARYT019099	409	46350.28	5.28	60.36	76.75	-0.60	nucl
PgbHLH25	GWHPARYT019460	461	48218.54	6.39	58.35	65.73	-0.47	nucl
PgbHLH26	GWHPARYT020023	447	49597.73	6.65	48.32	92.6	-0.27	cyto
PgbHLH27	GWHPARYT020118	311	33747.15	5.41	48.88	81	-0.37	nucl
PgbHLH28	GWHPARYT020199	223	25268.26	5.42	58.11	63.54	-0.90	nucl
PgbHLH29	GWHPARYT020515	381	40079.14	8.1	53.6	58.66	-0.58	chlo
PgbHLH30	GWHPARYT020731	150	16728.45	9.76	45.13	84.47	-0.36	pero
PgbHLH31	GWHPARYT012483	330	37213.06	6.61	51.3	73.3	-0.49	nucl
PgbHLH32	GWHPARYT012492	245	26889.53	5.59	50.12	77.67	-0.45	nucl
PgbHLH33	GWHPARYT012991	525	57087.86	5.16	58.67	70.99	-0.53	nucl
PgbHLH34	GWHPARYT013189	184	21226.79	7.87	40.66	63.59	-0.84	nucl
PgbHLH35	GWHPARYT013630	352	39743.05	7.12	47.1	74.23	-0.51	nucl
PgbHLH36	GWHPARYT014248	726	77521.83	6.61	52.43	60.15	-0.55	nucl
PgbHLH37	GWHPARYT014367	339	38222.76	5.63	55.3	77.91	-0.51	nucl
PgbHLH38	GWHPARYT014585	263	29128	6.26	53.33	90.49	-0.38	nucl
PgbHLH39	GWHPARYT009805	144	16686.28	9.34	36.11	92.78	-0.37	chlo
PgbHLH40	GWHPARYT009953	258	28931.53	5.86	62.16	84.57	-0.60	nucl
PgbHLH41	GWHPARYT010028	190	21766.15	9.77	43.1	82.05	-0.55	cyto
PgbHLH42	GWHPARYT010659	277	29964.32	6.36	47.55	58.16	-0.73	nucl
PgbHLH43	GWHPARYT010760	92	10516.79	9.09	86.33	91.2	-0.77	mito
PgbHLH44	GWHPARYT011115	247	26874.06	5.67	54.32	68.74	-0.63	nucl
PgbHLH45	GWHPARYT011701	258	29012.19	8.7	64.4	88.06	-0.43	nucl
PgbHLH46	GWHPARYT011714	678	74422.92	5.59	51.43	68.02	-0.59	nucl
PgbHLH47	GWHPARYT012095	244	27687.68	6.23	59.2	91.48	-0.53	nucl
PgbHLH48	GWHPARYT000483	280	31561.34	6.46	53.77	80.04	-0.33	cyto
PgbHLH49	GWHPARYT000502	340	38076.31	4.64	38.56	97.18	0.14	nucl
PgbHLH50	GWHPARYT000506	432	46661.33	5.83	48.12	55.37	-0.58	nucl
PgbHLH51	GWHPARYT000688	197	22247.21	7.13	35.28	82.13	-0.54	nucl
PgbHLH52	GWHPARYT001056	250	27827.11	6.23	71.81	61.96	-0.77	nucl
PgbHLH53	GWHPARYT001319	365	39864.96	4.81	69.4	80.16	-0.43	nucl
PgbHLH54	GWHPARYT001437	781	86353.11	5.29	47.31	76.88	-0.34	nucl
PgbHLH55	GWHPARYT001533	309	34201.96	5.68	54.91	61.55	-0.91	nucl
PgbHLH56	GWHPARYT001901	318	35266.01	6.01	52.14	67.45	-0.75	nucl
PgbHLH57	GWHPARYT001941	362	41153.38	5.08	51.98	73.23	-0.59	chlo
PgbHLH58	GWHPARYT001956	369	42184.95	8.57	59.25	81.08	-0.57	nucl
PgbHLH59	GWHPARYT001957	324	36186.82	5.96	50.22	79.17	-0.57	nucl
PgbHLH60	GWHPARYT001959	263	29563.91	6.54	45.18	91.9	-0.27	nucl
PgbHLH61	GWHPARYT001960	310	34657.68	5.87	44.14	92.13	-0.26	nucl
PgbHLH62	GWHPARYT001961	71	8026.21	6.1	58.91	80.99	-0.53	nucl

Gene name	Accession number	Number of Amino Acid	Molecular Weight	Theoretical pI	Instability Index	Aliphatic Index	Grand Average of Hydropathicity	Subcellular localization
PgbHLH63	GWHPARYT002155	355	38896.41	5.82	61.8	67.1	-0.65	nucl
PgbHLH64	GWHPARYT002167	222	24742.75	8.67	73.46	64.14	-0.41	nucl
PgbHLH65	GWHPARYT002379	330	36402.06	5.14	57.41	68.55	-0.77	nucl
PgbHLH66	GWHPARYT002564	229	25682.14	6.61	49.22	84.28	-0.67	nucl
PgbHLH67	GWHPARYT002569	91	10327.7	9.09	81.81	101.76	-0.51	cyto
PgbHLH68	GWHPARYT002888	260	28851.31	5.73	42.89	77.27	-0.51	nucl
PgbHLH69	GWHPARYT003195	254	28642.24	8.32	51.3	105.47	-0.15	chlo
PgbHLH70	GWHPARYT003196	293	33814.75	8.9	54.08	93.99	-0.41	golg
PgbHLH71	GWHPARYT003525	703	78588.64	8.9	46.97	85.59	-0.40	nucl
PgbHLH72	GWHPARYT007212	462	51748.94	7.02	55.66	59.74	-0.74	nucl
PgbHLH73	GWHPARYT007504	239	26171.67	8.54	53.06	75.19	-0.64	nucl
PgbHLH74	GWHPARYT007532	470	51475.56	6.26	64.77	55.17	-0.71	nucl
PgbHLH75	GWHPARYT009076	239	26944.86	8.67	52.29	97.03	-0.34	nucl
PgbHLH76	GWHPARYT009131	242	27530.38	8.77	59.15	92.56	-0.56	nucl
PgbHLH77	GWHPARYT009481	337	36960.73	5.3	55.96	78.19	-0.43	nucl
PgbHLH78	GWHPARYT014902	466	51768.38	6.47	57.88	57.79	-0.78	nucl
PgbHLH79	GWHPARYT015352	330	36722.14	4.99	36.15	71.18	-0.56	nucl
PgbHLH80	GWHPARYT015353	273	30003.15	9.36	43.3	68.97	-0.62	nucl
PgbHLH81	GWHPARYT015406	226	24983.86	4.59	48.24	71.55	-0.42	nucl
PgbHLH82	GWHPARYT015663	159	18166.84	10.22	44.72	78.43	-0.56	nucl
PgbHLH83	GWHPARYT015939	535	59712.58	6.36	62.51	87.33	-0.30	nucl
PgbHLH84	GWHPARYT016155	719	80994.64	4.83	67.58	77.58	-0.59	nucl
PgbHLH85	GWHPARYT016324	562	61368.34	6.59	54.2	64.95	-0.71	nucl
PgbHLH86	GWHPARYT016428	527	57452.75	5.43	62.28	58.12	-0.60	nucl
PgbHLH87	GWHPARYT016568	636	70902.11	5.83	45.86	78.33	-0.37	nucl
PgbHLH88	GWHPARYT016593	480	53486.17	6.45	52.08	76.73	-0.53	nucl
PgbHLH89	GWHPARYT016625	297	31277.35	5.91	49.98	74.28	-0.33	nucl
PgbHLH90	GWHPARYT016724	291	32873.78	9.79	53.48	84.4	-0.52	nucl
PgbHLH91	GWHPARYT020781	437	48793.42	7.25	68.12	81.46	-0.60	nucl
PgbHLH92	GWHPARYT020834	395	43663.33	5.92	37.61	83.62	-0.40	nucl
PgbHLH93	GWHPARYT020854	623	68556.8	6.56	41.68	76.55	-0.49	nucl
PgbHLH94	GWHPARYT021416	191	20787.35	5.64	49.67	86.28	-0.44	nucl
PgbHLH95	GWHPARYT021574	238	27172.25	5.83	65.45	66.85	-0.85	nucl

Table S2. Ka/Ks values of duplicated *PgbHLH* gene pairs.

Seq_1	Seq_2	Ka	Ks	Ka_Ks	Effective Len	AverageS-sites	Average N-sites	cN	cS	pN	pS
GWHPARYT004930	GWHPARYT002379	0.447	1.788	0.250	972	213.583	758.417	255.583	145.417	0.337	0.681
GWHPARYT005249	GWHPARYT002167	0.336	3.455	0.097	627	136.250	490.750	132.833	101.167	0.271	0.743
GWHPARYT005260	GWHPARYT002155	0.443	2.621	0.169	789	181.167	607.833	203.250	131.750	0.334	0.727
GWHPARYT005453	GWHPARYT001941	0.311	1.649	0.189	900	193.833	706.167	179.750	129.250	0.255	0.667
GWHPARYT004355	GWHPARYT006262	0.538	1.705	0.315	1230	272.750	957.250	367.500	183.500	0.384	0.673
GWHPARYT006262	GWHPARYT012991	0.481	2.118	0.227	1206	270.500	935.500	332.167	190.833	0.355	0.705
GWHPARYT006549	GWHPARYT010028	0.309	NaN	NaN	492	106.833	385.167	97.500	81.500	0.253	0.763
GWHPARYT019017	GWHPARYT016324	0.356	NaN	NaN	1509	337.167	1171.833	331.917	254.083	0.283	0.754
GWHPARYT020118	GWHPARYT016625	0.321	1.769	0.181	846	205.917	640.083	167.167	139.833	0.261	0.679
GWHPARYT004355	GWHPARYT012991	0.308	1.977	0.156	1467	335.000	1132.000	285.750	233.250	0.252	0.696
GWHPARYT004700	GWHPARYT010659	0.254	1.351	0.188	795	179.667	615.333	132.500	112.500	0.215	0.626
GWHPARYT004757	GWHPARYT010760	0.122	1.360	0.090	270	61.333	208.667	23.500	38.500	0.113	0.628
GWHPARYT010760	GWHPARYT002569	0.185	2.149	0.086	273	62.917	210.083	34.500	44.500	0.164	0.707
GWHPARYT012095	GWHPARYT003195	0.575	3.865	0.149	711	158.583	552.417	221.750	118.250	0.401	0.746
GWHPARYT000483	GWHPARYT007504	0.302	1.821	0.166	711	163.417	547.583	136.250	111.750	0.249	0.684
GWHPARYT000506	GWHPARYT007532	0.406	3.056	0.133	1182	268.000	914.000	286.417	197.583	0.313	0.737
GWHPARYT004766	GWHPARYT002564	0.494	1.837	0.269	672	151.167	520.833	188.417	103.583	0.362	0.685
GWHPARYT004757	GWHPARYT002569	0.147	1.477	0.099	267	61.333	205.667	27.417	39.583	0.133	0.645
GWHPARYT004355	GWHPARYT006262	0.538	1.705	0.315	1230	272.750	957.250	367.500	183.500	0.384	0.673
GWHPARYT006262	GWHPARYT012991	0.481	2.118	0.227	1206	270.500	935.500	332.167	190.833	0.355	0.705
GWHPARYT004355	GWHPARYT012991	0.308	1.977	0.156	1467	335.000	1132.000	285.750	233.250	0.252	0.696
GWHPARYT004700	GWHPARYT010659	0.254	1.351	0.188	795	179.667	615.333	132.500	112.500	0.215	0.626
GWHPARYT004757	GWHPARYT010760	0.122	1.360	0.090	270	61.333	208.667	23.500	38.500	0.113	0.628
GWHPARYT006549	GWHPARYT010028	0.309	NaN	NaN	492	106.833	385.167	97.500	81.500	0.253	0.763
GWHPARYT005249	GWHPARYT002167	0.336	3.455	0.097	627	136.250	490.750	132.833	101.167	0.271	0.743
GWHPARYT005260	GWHPARYT002155	0.443	2.621	0.169	789	181.167	607.833	203.250	131.750	0.334	0.727
GWHPARYT005453	GWHPARYT001941	0.311	1.649	0.189	900	193.833	706.167	179.750	129.250	0.255	0.667
GWHPARYT004757	GWHPARYT002569	0.147	1.477	0.099	267	61.333	205.667	27.417	39.583	0.133	0.645
GWHPARYT004766	GWHPARYT002564	0.494	1.837	0.269	672	151.167	520.833	188.417	103.583	0.362	0.685
GWHPARYT004930	GWHPARYT002379	0.447	1.788	0.250	972	213.583	758.417	255.583	145.417	0.337	0.681
GWHPARYT019017	GWHPARYT016324	0.356	NaN	NaN	1509	337.167	1171.833	331.917	254.083	0.283	0.754
GWHPARYT020118	GWHPARYT016625	0.321	1.769	0.181	846	205.917	640.083	167.167	139.833	0.261	0.679
GWHPARYT012095	GWHPARYT003195	0.575	3.865	0.149	711	158.583	552.417	221.750	118.250	0.401	0.746
GWHPARYT010760	GWHPARYT002569	0.185	2.149	0.086	273	62.917	210.083	34.500	44.500	0.164	0.707
GWHPARYT000483	GWHPARYT007504	0.302	1.821	0.166	711	163.417	547.583	136.250	111.750	0.249	0.684
GWHPARYT000506	GWHPARYT007532	0.406	3.056	0.133	1182	268.000	914.000	286.417	197.583	0.313	0.737
